# Overexpression of CD97 in intestinal epithelial cells attenuates LPS-induced pro-inflammatory cytokine induction via stabilization of β-catenin early in life

**DOI:** 10.1371/journal.pone.0354507

**Published:** 2026-07-30

**Authors:** Niklas Dressler, Steffi Mayer, Thomas Wiemers, Florentine Weise, Xiaoyan Feng, Gabriela Aust, Nicole Peukert, Martin Lacher, Jan Riedel

**Affiliations:** 1 Department of Paediatric Surgery, University Hospital Leipzig, Germany; 2 Department of Visceral, Transplantation, Thoracic and Vascular Surgery, University of Leipzig, Germany; University of Illinois at Chicago, UNITED STATES OF AMERICA

## Abstract

Acute inflammatory conditions in the intestine of preterm infants are linked to increased susceptibility due to the immature degree of the gut’s epithelial barrier, microbiota, and pattern recognition receptors. Toll-like receptor 4 (TLR4) plays a pivotal role in recognizing lipopolysaccharides (LPS) from gram-negative bacteria, triggering pro-inflammatory cytokine responses (TNF-α, IL-1β, CXCL1) via nuclear factor kappa B (NF-κB) signaling. These processes are implicated in necrotizing enterocolitis (NEC), a severe gastrointestinal condition in premature infants. CD97, an adhesion G-protein coupled receptor (aGPCR), has emerged as a modulator of immune responses influencing inflammatory signaling pathways. CD97 expression is typically low in intestinal epithelial cells (IECs); however, protective effects of increased CD97 levels have been described in experimentally induced colitis. In this study, we examined the role of CD97 in modulating the inflammatory response in the immature gut, utilizing wild-type (WT) and transgenic CD97-overexpressing mice (TgCD97) with epithelial-specific expression in the intestinal epithelium. LPS was administered to IECs and organ segments isolated from the small intestine of mice of specific ages, modeling certain stages of human perinatal/postnatal gut development. CD97 overexpression attenuated LPS-induced TNF-α expression in IECs during early intestinal development while IECs from adult mice remained unaffected. This effect was attributed specifically to ileal tissue. Attenuation was mediated by β-catenin stabilization, leading to suppressed LPS/NF-κB signaling. Inhibition of β-catenin in TgCD97 IECs reversed the anti-inflammatory phenotype restoring pro-inflammatory gene expression. These findings suggest that CD97 overexpression modulates the inflammatory response in the developing gut by stabilizing β-catenin and interacting with the LPS/NF-κB axis. This effect was restricted to early developmental stages and ileal tissue, highlighting a previously unrecognized role of CD97 in age-dependent endotoxin tolerance. These findings identify a novel CD97-β-catenin-NF-κB regulatory axis in the immature small intestine and highlight its potential as a therapeutic target to protect the immature neonatal gut from inflammatory damage.

## Introduction

The pathological mechanisms of acute inflammatory conditions in the immature intestine, especially in preterm neonates, are not yet fully understood. Compared to adults, the immature intestine is more sensitive to inflammatory stress. This is due to an age-dependent elevated presence and activity of distinct pattern-recognition receptors and signaling pathways during early development, particularly in the case of preterm birth [1–3]. However, an adequate response to ubiquitous commensal bacterial epitopes and endotoxin is crucially required for uncompromised tissue development and acquisition of the epithelial barrier’s physiological endotoxin tolerance over the course of gut maturation [4].

Intestinal immaturity plays a pivotal role in the development of gastrointestinal diseases, and Toll-like receptor 4 (TLR4), expressed on intestinal epithelial cells (IECs), is crucial in this process. TLR4 recognizes lipopolysaccharides (LPS) derived from gram-negative bacteria and induces a pro-inflammatory cellular response primarily mediated by the phosphorylation of the nuclear factor kappa B (NF-κB) subunit p65. Activated p65 translocates from the cytoplasm to the nucleus, inducing the transcription of pro-inflammatory genes such as *TNF-α, IL-1β,* and *CXCL1* [5]. Subsequent epithelial cell apoptosis and increased mucosal permeability result in epithelial barrier damage and bacterial infiltration of deeper tissues [6]. Mechanisms counteracting TLR4/p65 signaling have been shown to reduce intestinal pro-inflammatory conditions [7]. These processes are highly relevant in the context of necrotizing enterocolitis (NEC), a severe gastrointestinal inflammatory disease primarily affecting preterm infants, particularly in the terminal ileum. In NEC, TLR4 plays a central role in this condition, as disrupted TLR4/p65 signaling has been shown to protect against disease development and progression [6,7]. Although TLR4/NF-κB activation is critical for host immunity and defense against pathogens, an exaggerated and prolonged NF-κB activation in premature infants leads to an excessive pro-inflammatory response. This results in elevated cytokine levels, contributing to intestinal tissue injury, as seen in NEC [8]. Having identified the relevance of TLR4/NF-κB signaling further studies identifying regulators and novel pathways interacting with this axis are needed

The adhesion G-protein coupled receptor (aGPCR) CD97 has been increasingly studied for its role in the innate immune and epithelial pro-inflammatory signaling [9–11]. CD97 is ubiquitously expressed on the surface of lymphocytes, monocytes, macrophages, granulocytes, and dendritic cells, as well as mesenchymal cells and smooth muscle cells. In the intestinal epithelium, CD97 expression is low under normal conditions, while present in the stroma of the villi and crypts [12,13]. It acts as a mediator in the immune defense regulating the activation status of T-lymphocytes and the migration of neutrophils and monocytes to sites of inflammation [10,11]. Likewise, overexpression of CD97 has been shown to inhibit the extent of pro-inflammatory gene expression in experimental dextran sulfate sodium (DSS)-induced colitis in adult mice [14]. This effect was mediated by inactivation of glycogen synthase kinase 3β (GSK-3β) and subsequent stabilization of junctional β-catenin, strengthening the structural integrity of E-cadherin-based adherens junctions. In macrophages, CD97 has been shown to negatively regulate LPS-induced p65 activation by upregulation of peroxisome proliferator-activated receptor gamma (PPAR-γ) [15]. While these studies indicate CD97 as a potential candidate to modulate intestinal LPS/TLR4/p65 signaling in neonates, data from small intestinal tissues and immature gut samples are still lacking [15].

In this study, we aimed to further characterize the effect of CD97 on the inflammatory response of the immature small intestinal epithelium to endotoxin stress. Using a transgenic CD97-overexpressing mouse model (TgCD97) with epithelial-specific expression, we isolated IECs from the small intestines of mice at distinct postnatal stages and subjected them to bacterial LPS. These stages correspond to key phases of human gut development, characterized by distinct gene expression patterns of structural and differentiation proteins, as well as tissue maturation levels [16]. This approach enabled a comprehensive evaluation of the hypothesized beneficial effects of CD97 overexpression on the neonatal inflammatory response, and facilitated the identification of potential mediators and therapeutic targets involved in the age-dependent acquisition of endotoxin tolerance.

## Methods and materials

### Animals

Wild type (WT) C57BL/6 mice on postnatal day (P) 7, 14, 28 and 56 were purchased from the animal care facility of the medical faculty of Leipzig (MEZ). The animals were housed under specific pathogen-free conditions in strict accordance with local animal protection legislation. Investigated age groups of mice were selected based on the work of Stanford et al., which identified parallels in the maturation timelines of intestinal tissues between mice and humans by comparing the expression levels of key genes involved in homeostasis, for example MKI67, BAX/BCL2, LGR5, BMI1, ErbB receptors, and structural components, e.g., CDH1, TJP1. Specifically, P7 murine intestines corresponded to fetal human gut tissue, P14 to preterm neonatal human intestinal tissue, P28 to term-born human intestines, and P56 to adult human gut tissue [17].

Transgenic mice overexpressing CD97 specifically in IECs under the control of the villin promoter (C57BL/6J-Tg (villin-mCD97(4EGF)), referred to as the TgCD97 group, and CD97 knock out mice (C57BL/6J CD97^-/-^, referred to as KO CD97 group) were provided by G. Aust. The generation protocol, characteristics and previous descriptions of both animal strains can be found in prior studies [14,18]. In brief, for the generation of TgCD97 mice, a 9kb regulatory region of the mouse villin gene was place upstream of a CD97 cDNA to generate a villin-CD97 expression construct. Pronuclear microinjection of the purified expression construct into fertilized oocytes of C57BL/6J mice was performed to generate Tg mice. Founders were mated to wild-type mice [14]. CD97 knockout mice were generated using Cre/*lox*P-mediated DNA recombination. The targeting construct consisted of a *lox*P-PGK*neo*-*lox*P cassette which was inserted upstream of exon 2 and a third loxP site was placed downstream of exon 5 of the murine CD97 gene spanning intron 1 to exon 12. The linearized targeting construct was electroporated into 129/Ola-derived embryonic stem (ES) cells. Selection with G418 was performed for 8 days and G418-resistant ES cell clones were used to PCR based verification of homologous recombination. Subsequently clone IIH10 was microinjected into C57BL/6J-derived blastocytes to generate chimeric offspring which were mated with C57BL/6J mice. Mice positive for germline transmission were mated with homozygous EIIa*Cre* mice expressing Cre recombinase during embryonic development. CD97 deficient mice were backcrossed on a C57BL/6J background [18].

Animal care and experimental procedures were approved by the institutional review board (State Directorate Saxony, Leipzig, Germany; Proposal T02/20;T06/21). Age-matched WT C57BL/6J mice served as controls for all experiments.

### Animal welfare statement

Animals were euthanized solely for tissue collection and no experimental procedures were performed on living animals prior to organ harvest. Mice up to two weeks of age were euthanized by decapitation, whereas mice older than two weeks were euthanized by cervical dislocation, in accordance with institutional and national regulations.

As the study involved only post-mortem tissue collection, no anesthesia or analgesia was required. All procedures were performed by trained personnel, and every effort was made to minimize animal suffering and distress. Organs were collected immediately following confirmation of death.

### Antibodies and reagents

Rabbit monoclonal antibodies specific for phospho-NF-κB p65 (Ser536) (93H1) were procured from Cell Signaling Technology (Boston, USA). Rabbit polyclonal anti-β-actin antibody was obtained from Abcam (Cambridge, England). HRP-conjugated secondary antibodies were sourced from Jackson Immunoresearch (Philadelphia, USA). Lipopolysaccharide (LPS) derived from *E. coli* (O127:B8) was supplied by Sigma Aldrich (Hamburg, Germany). Protease and phosphatase inhibitors were purchased from Serva (Heidelberg, Germany). The β-catenin signaling inhibitor, iCRT3, was provided by Selleckchem (Houston, USA).

### Isolation and cultivation of primary intestinal epithelial cells

Mice of specific age groups were sacrificed, and the small intestines were removed, washed several times with ice-cold PBS and opened longitudinally. The intestines were cut into 1–2 mm pieces and incubated for 10 minutes at 37°C in Ca^2+^- and Mg^2+^-free HBSS (Gibco; Thermo Fisher, Waltham, USA) containing 10% FCS (Sigma-Aldrich), 1% Pen/Strep (Gibco), and 100 mM DTT (Thermo Fisher) under constant shaking. Cellular aggregates were removed by filtration through 100 µm and 30 µm mesh (Milteny Biotech, Bergisch Gladbach, Germany). Cells were harvested by centrifugation at 270g for 10 minutes at 4°C and resuspended in DMEM (Gibco) supplemented with 10% FCS, 100 mM pyruvate (Gibco), 100 mM glutamine (Gibco), and 1% Pen/Strep. Viable cell count was determined using a Neubauer counting chamber with Trypan Blue staining. Viable IECs were morphologically identified with an approximate viability rate of 80% per experiment and seeded in uncoated 24-well plates at a density of 5x10^5^ cells/well. Cells were maintained under standard cell culture conditions (37°C, 5% CO₂). Two hours after seeding, cells were stimulated with 1 µg/mL LPS for 3h. Preliminary experiments evaluating various durations of LPS incubation (0.5, 1, 3, 6, 12, and 24 hours) identified 3 hours as the optimal time point, corresponding to peak mRNA and protein responses. For experiments using β-catenin signaling inhibitor (iCRT3), TgCD97 IECs were preincubated with 25 µM of iCRT3 for 2 hours and subsequently stimulated with 1 µg/mL LPS for 3h. WT control database used in this study were generated as part of the same experimental series and have been partially reported as previously. These data were reused to ensure direct comparability between experimental groups under the same experimental conditions [17].

### Isolation and cultivation of small intestine segment explants

Two-week-old mice were sacrificed, and the small intestines were removed and were washed several times in ice-cold PBS. The intestine was divided into 2 cm segments from the terminal ileum and central jejunum. These segments were opened longitudinally and placed on ThinCert® inserts (Greiner) in CellStar® 6-well plates (Greiner), with the epithelial layer facing the surface of the insert. The segments were immediately stimulated with 1 µg/ml LPS.

### RNA isolation, cDNA synthesis and Real-Time PCR

RNA extraction was conducted using the RNeasy Micro Kit (Qiagen, Hilden, Germany) according to the manufacturer´s instructions First-strand cDNA synthesis was accomplished by M-MLV Reverse Transcriptase Kit (Invitrogen). Quantitative real-time PCR (RT-qPCR) analysis was carried out using the Mastercycler Realplex 2 (Eppendorf) and the QuantiTect SYBR® Green PCR Kit (Qiagen). Gene specific oligonucleotides were obtained from Biomers (Ulm, Germany) (Table 1).

**Table 1:** Oligonucleotide sequences used for RT-qPCR.

Target	Forward	Reverse
*Gapdh*	*TGAAGCAGGCATCTGAGGG*	*CGAAGGTGGAAGAGTGGGAG*
*β-actin*	*CCACAGCTGAGAGGGAAATC*	*TCTCCAGGGAGGAAGAGGAT*
*Tnf-⍺*	*TTCCGAATTCACTGGAGCCTCGAA*	*TGCACCTCAGGGAAGAATCTGGAA*
*β-catenin*	*CGAGGACTCAATACCATTCC*	*CTGAGCAAGTTCACAGAGG*
*Tlr4*	*GCTTACACCACCTCTCAAAC*	*CAGCCACCAGATTCTCTAAAC*
*Cd97*	*GGGAAGAGTACTGGAAATGG*	*TCACATCCCTGATTCTGAGG*
*Cxcl1*	*ACCCAAACCGAAGTCATAGCC*	*TTGTCAGAAGCCAGCGTTCA*
*Il-1β*	*TGGACCTTCCAGGATGAGGACA*	*GTTCATCTCGGAGCCTGTAGTG*

### Immunoblot analysis

Cells were lysed in RIPA buffer supplemented with a protease inhibitor mix (Merck Milipore, Darmstadt, Germany) and phosphatase inhibitor mix (Serva). For cytoplasmatic and nuclear protein isolation, cells were lysed in CSK-T buffer (10 mM HEPES-KOH pH7.4, 300 mM sucrose, 100 mM NaCl, 2 mM MgCl_2_, 0.5% Triton X-100) and incubated on ice for 10 minutes. Cytoplasmatic and nuclear fractions were separated by centrifugation at 5000g for 5 min. The supernatant containing cytoplasmatic protein was transferred to another tube and centrifuged again at 18000g for 15 min. Cell nuclei in the pellet fraction were washed three times in CSK-T buffer and lysed using RIPA buffer for 30 min on ice.

Protein quantification was performed with Pierce™ BCA Protein Assay Kit (ThermoFisher). Equal amounts of proteins were separated by SDS-PAGE and transferred to PVDF membranes (Merck Milipore). Membranes were blocked in 5% non-fat dry milk dissolved in TBS-T and probed with specific primary antibodies overnight at 4°C, followed by incubation with HRP-conjugated secondary antibodies. Detection was performed using WesternBright Sirius ECL Kit (Advansta, Menlo Park, CA) on a ChemStudio Touch 815 Imager (Analytik Jena, Jena, Germany). Densiometric quantification of western blot data was performed with Image J (Image J 1.54r)

### Statistical analysis

Data were analyzed using GraphPad Prism Version 9. Normal distribution and consistency were evaluated using Shapiro-Wilk test (α < 0.05), Grubbs’ test (α < 0.05), and ROUT method (Q < 0.01). Parametric data were analyzed using two-tailed unpaired t-tests and two-way ANOVA, while non-parametric data were analyzed using the Mann-Whitney U test. Linear regression analysis was performed as indicated in the figure legends. Statistical significance was defined as *p < 0.05, **p < 0.01, and ***p < 0.001. All graphs are shown as mean ±SD from at least three independent biological experiments.

## Results

### CD97 overexpression in IECs attenuates the LPS-induced Tnf-α expression during early stages of small intestinal development.

**Fig 1:**
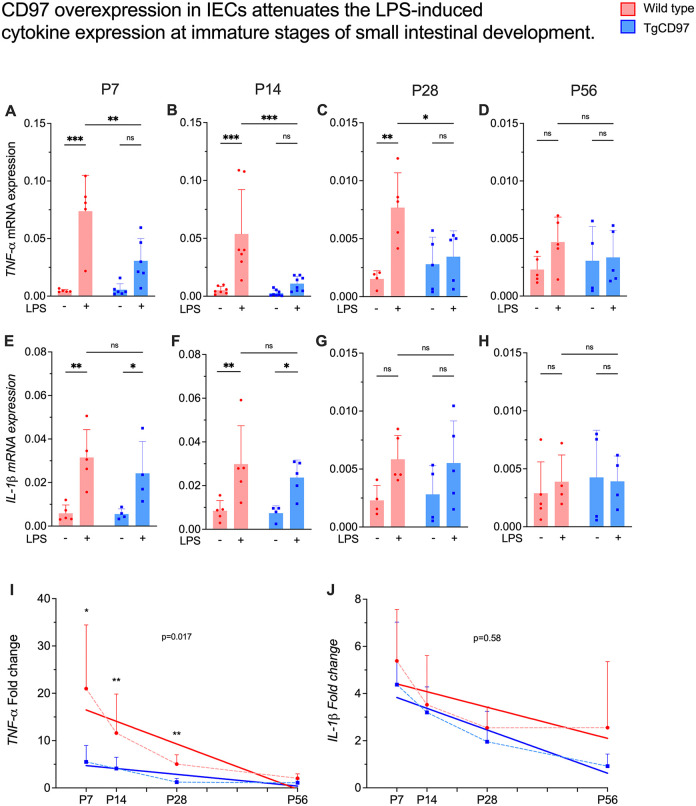
CD97 overexpression in IECs attenuates the LPS-induced *Tnf-α* expression during early stages of small intestinal development. Primary IECs of WT and TgCD97 mice at different stages of gastrointestinal development were isolated and stimulated with 1 µg/mL LPS for 3 hours. mRNA was then isolated and gene expression for *Tnf-α* (A-D) and *Il-1β* (E-H) was analyzed by RT-qPCR, using β-actin as reference gene for normalization. The ages of mice were: P7 = 1 week, P14 = 2 weeks, P28 = 4 weeks, P56 = 8 weeks. Statistical analysis was performed using two-way-ANOVA compare between genotype (WT vs. TgCD97) and stimulation conditions (-/ + LPS). The fold change of *Tnf-α* (I) and *Il-1β* (J) expression following LPS-stimulation was calculated. A two-tailed unpaired T-test was used to compare the fold changes between WT and TgCD97 within the respective age groups. Linear regression analysis was conducted to determine statistical difference between the curves. WT control data have been partially published previously and are shown here for direct comparison [17]. Results are presented as mean ± SD (n = 4–9; *p < 0.05; **p < 0.01, ***p < 0.001; ns = not significant).

To characterize the impact of CD97 overexpression in small IECs under inflammatory conditions, we isolated primary murine IECs from wild-type (WT) and TgCD97 animals and analyzed the expression of pro-inflammatory genes following LPS stimulation. To account for differences during gastrointestinal maturation, we selected primary IECs at distinct stages, which have been previously shown to represent phases of human intestinal development [16]. When comparing Tnf-α expression between unstimulated and LPS-stimulated WT IECs, we observed a significant increase up to four weeks of age (Fig 1A-C). In contrast, TgCD97 IECs showed no comparable significant LPS-induced Tnf-α expression (Fig 1A-C). Moreover, LPS-stimulated TgCD97 IECs showed a significantly reduced Tnf-α expression compared to LPS-stimulated WT IECs at P7, P14 and P28. By eight weeks of age, neither WT nor TgCD97 IECs showed significant Tnf-α upregulation in response to LPS compared to controls (Fig 1D). Over the course of postnatal gastrointestinal development, LPS-stimulated Tnf-α induction was significantly lower in TgCD97 IECs compared to WT IECs (Fig 1I).

For IL-1β, differences were detected between LPS-stimulated WT and TgCD97 IECs in age groups P7 and P14, but no direct comparison reached statistical significance (Fig 1E-H). Similar to findings in Tnf-α response, LPS-induced Il-1β expression in WT and TgCD97 decreased over the course of intestinal development (Fig 1J).

### CD97-mediated Tnf-α attenuation is restricted to LPS-responsive ileal tissue

**Fig 2:**
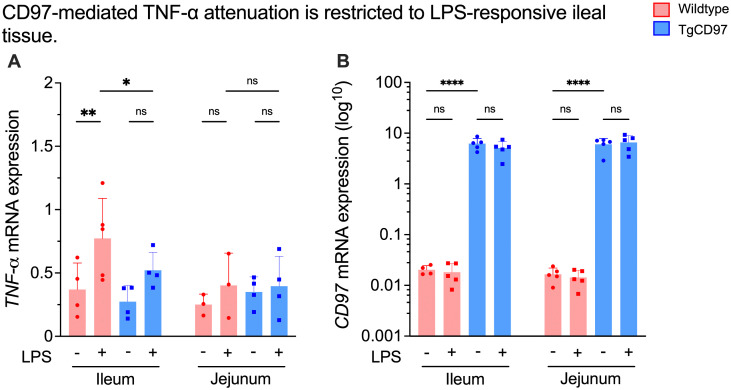
CD97-mediated attenuation of *Tnf-α* is restricted to LPS-responsive ileal tissue. Intestine from P14 mice were isolated, and whole-tissue segment explants from ileum and jejunum were stimulated with 1 µg/mL LPS for 3 hours. Gene expression analysis of (A) Tnf-α and (B) CD97 was determined via RT-qPCR using *β-actin* as reference gene. Two-way-ANOVA was conducted to compare the genotypes (WT vs. TgCD97) and stimulation conditions (- / + LPS) in each tissue segment. Results are presented as mean ± SD (n = 3–5, * p < 0.05, **p < 0.01, ns = not significant).

To verify our findings from IEC single-cell cultures, we next assessed the gene expression of inflammatory markers in murine intestines at the tissue level. Since human NEC mainly affects the terminal ileum of premature infants, we selected the ileum and jejunum of two-week-old mice, which resemble the intestinal tissue of preterms [16,19]. Consistent with our cellular findings, LPS induced an increase in *Tnf-α* gene expression in ileal segments of WT that was not present in TgCD97-derived ileal tissue (Fig 2A). Tissue explants from the jejunum of both WT and TgCD97 remained unresponsive to LPS stimulation (Fig 2A). We ruled out differences of CD97 receptor expression between segments as main conveyor of this effect: CD97 expression was comparable between the jejunum and ileum in wild-type (WT) tissues. As expected, CD97 expression was significantly higher in both the jejunum and ileum of TgCD97 mice compared to WT mice. Moreover, no significant alteration in Cd97 gene expression was observed following LPS exposure in either genotype or tissue segment. (Fig 2B). Differences between the segments at the tissue level were not further investigated as we proceeded to characterize the effects of CD97 and its molecular mediators at the intestinal epithelial cell level.

### CD97 knockout does not affect LPS-mediated Tnf-α gene expression

**Fig 3:**
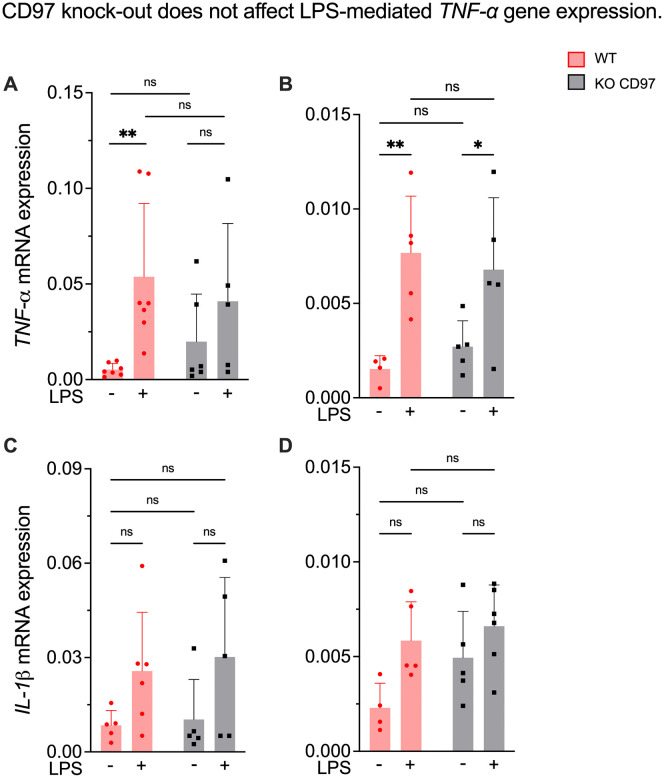
CD97 knockout does not affect LPS-mediated *Tnf-α* gene expression. Primary IECs from WT and KO CD97 mice were isolated at P14 (A&C) and P28 (B&D) of age and stimulated with 1 µg/mL LPS for 3 hours. Pro-inflammatory gene expression of *Tnf-α* (A&B) and *Il-1β* (C&D) was determined by RT-qPCR. *β-actin* was used as reference gene. Two-way-ANOVA was performed for statistical analysis to compare between genotype (WT and KO CD97) and stimulation (-/ + LPS). WT control data shown in this figure are identical to those presented in Fig 1 and were derived from the same experimental dataset which have been partially published previously and are shown here for direct comparison [17]. Results are presented as mean ± SD (n = 5–7, ns = not significant).

To assess if lack of CD97 resulted in a proportionally amplified cytokine induction, we analyzed LPS-induced gene expression between WT and KO CD97 IECs during intestinal development at two and four weeks. The gene expression level of *Tnf-α* in KO CD97-derived IECs were comparable to those in WT IECs at both stages of maturation, with no statistical differences observed (Fig 3A, B). Similarly, the expression of *Il-1β* showed no difference between KO CD97 and WT IECs at either time point (Fig 3C, D). This somewhat unexpected result prompted us to focus on the molecular mediators downstream of the aGPCR.

### CD97 overexpression suppresses NF-κB activation and promotes LPS-tolerance, which is associated with β-catenin stabilisation

**Fig 4:**
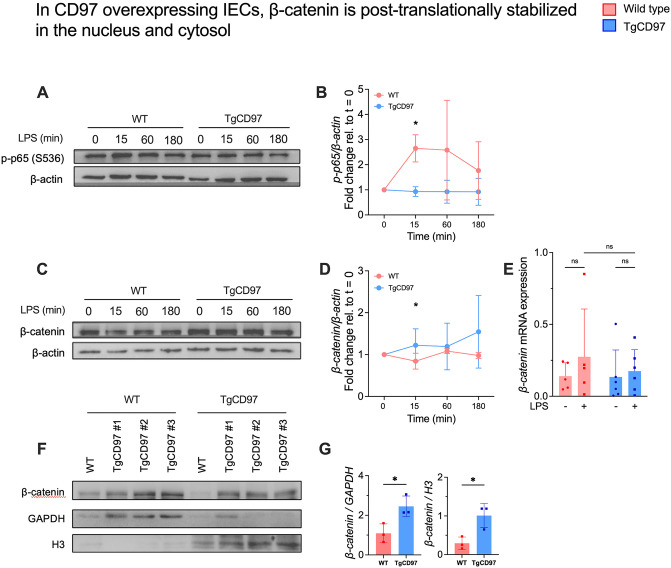
CD97 overexpression suppresses NF-κB activation and is associated with ⁠ post-translational stabilized β-ca⁠tenin. (A) Representative western blot analysis of phosphorylated p65 (S536) over the course of time after stimulation with 1 µg/mL LPS in P14 IECs from WT and TgCD97. (B) Densiometric quantification of phosphorylated p65 (S536) related to β-actin over the course of time. Values are depicted as Fold change related to t = 0 (n = 3). (C) Representative western blots for analysis of β-catenin over the course of time after stimulation with 1 µg/mL LPS in P14 IECs from WT and TgCD97. (D) Densiometric quantification of β-catenin related to β-actin over the course of time. Values are depicted as Fold change related to t = 0 (n = 3). (E) RT-qPCR analysis of β-catenin transcripts in P14 IECs from WT and TgCD97 stimulated with 1 µg/mL LPS (n = 5–6). (F) Representative western blot for the distribution of β-catenin from one P14 WT and three P14 TgCD97 (TgCD97 #1, #2, #3) within the cytosolic and nuclear fraction. (G) Densiometric quantification of cytosolic β-catenin between WT and TgCD97 IECs. Cytosolic β-catenin levels were related to GAPDH (n = 3). (H) Densiometric quantification of nuclear β-catenin between WT and TgCD97 IECs. Nuclear β-catenin was related to H3 (n = 3). Statistical analysis of gene expression data was performed using Two-way-ANOVA and paired t-test was used for analysis of densiometric western blot data. (* = p < 0.05, ns = not significant). Graph shown as mean ± SD.

We assessed the activation status of the NF-κB subunit p65. In IECs derived from WT we observed an increase in the phosphorylation status of p65 after 15 minutes following LPS stimulation, while we did not detect a similar increase in TgCD97 IECs (Fig 4A). Densiometric quantification of phosphorylated p65 revealed a significant difference between WT and TgCD97 after 15 minutes of LPS treatment (Fig 4B). The overall level of phosphorylated p65 in TgCD97 IECs remained stable throughout the LPS challenge. WT p-p65 levels started to recovered back to unstimulated levels at three hours after endotoxin challenge.

A main characteristic of the TgCD97 mouse model is an increased epithelial integrity mediated by increase of adherens junction stability from membrane-bound β-catenin through GSK-3β inactivation [14]. In conditions of severe intestinal inflammation, LPS signaling leads to a decrease in β-catenin via an increase in GSK-3β activation [20].

Thus, we investigated the protein quantity of β-catenin in WT and TgCD97 IECs from two-week-old mice following LPS stimulation (Fig 4C). In WT IECs, LPS-induced TLR4 signaling led to a decrease in the total amount of β-catenin after 15 minutes. In contrast, TgCD97 IECs exhibited an overall increase in β-catenin levels with no detectable influence of LPS administration, as observed in WT IECs. Densiometric quantification of β-catenin revealed a significant difference between WT and TgCD97 at 15 minutes of LPS treatment (Fig 4D). mRNA levels of *β-cateni*n were equal between genotypes and remained unaffected by LPS (Fig 4E) excluding transcriptional regulation of β-catenin mRNA as main conveyor of this effect.

To determine if the stabilized β-catenin in TgCD97 was more abundant in the cytosolic or potentially transcriptionally active nuclear form, extracts of both compartments were analyzed separately. In WT IECs, baseline levels of β-catenin were low and exclusively found in the cytosolic compartment (Fig 4F), whereas the stabilized β-catenin in TgCD97 IECs was highly elevated in both cytosolic and nuclear extracts (Fig 4F). Densiometric quantification of cytosolic and nuclear β-catenin levels revealed a significant difference between WT and TgCD97 derived IECs (Fig 4G).

### Inhibition of β-catenin signaling increases LPS-induced pro-inflammatory gene expression in TgCD97 IECs

**Fig 5:**
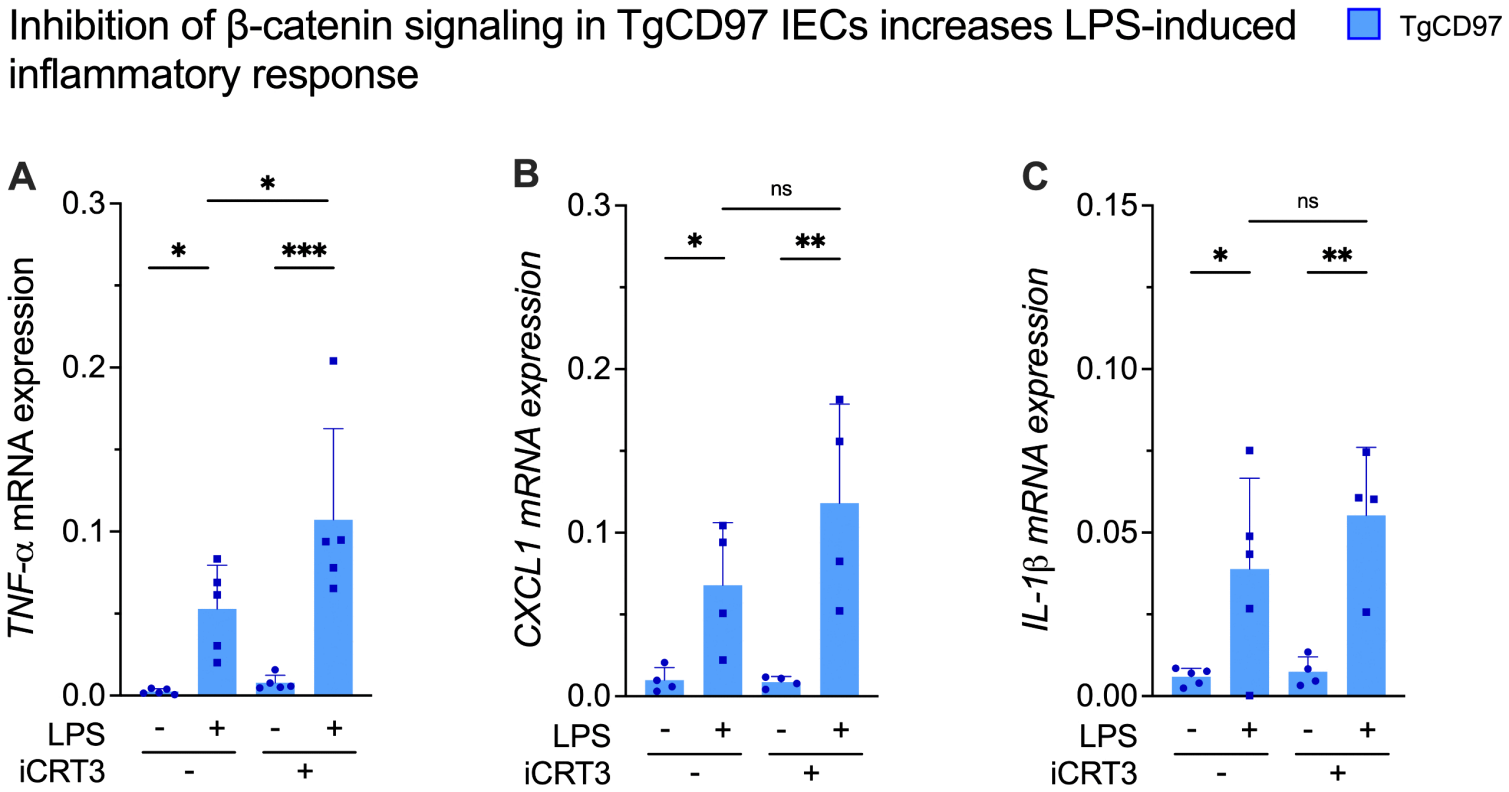
Interfering with β-catenin signaling increases LPS-induced pro-inflammatory gene expression in TgCD97 IECs. IECs from P14 WT and TgCD97 mice were isolated and pre-treated with 25µM iCRT3 for 2 hours. Subsequently, cells were stimulated with 1 µg/mL LPS for 3 hours. Gene expression of *Tnf-α* (A), *Il-1β* (B) and *Cxcl1* (C) was determined by RT-qPCR. Two-way ANOVA was performed for statistical analysis. (n = 4–5, *p < 0.05, **p < 0.01, ***p < 0.001, ns = not significant). All graphs are shown as mean ± SD.

We next sought to determine whether we could reverse suppressing effects of β-catenin stabilization via CD97 overexpression on cytokine expression by blocking β-catenin signaling. We inhibited the downstream activity of β-catenin in TgCD97 by administering the commercial Wnt/β-catenin signaling inhibitor iCRT3 [20]. Application of iCRT3 restored a significant pro-inflammatory response as assessed by gene expression of *Tnf-α* (Fig 5A), *Il-1β* (Fig 5B) and *Cxcl1* (Fig 5C) after LPS stimulation of TgCD97 IECs from two-week-old mice.

Thus, the attenuated pro-inflammatory gene expression after LPS stimulation in TgCD97 IECs might be attributed to the increased and stabilized protein expression of β-catenin in cytosol and nucleus of these enterocytes.

## Discussion

In the current study, we investigated the impact of CD97 overexpression on the pro-inflammatory response to LPS in small IECs at various stages of intestinal development While CD97 has previously been implicated in the regulation of LPS-induced inflammatory signaling in immune, its role in the immature small intestinal epithelium has not been defined. We observed an overall reduced inflammatory response in TgCD97 IECs particularly at early stages of gastrointestinal development. This effect was not directly mediated by the overexpression of CD97 itself but was mediated through CD97’s role in post-translational stabilization of β-catenin and suppression of LPS-induced NF-κB activation. Inhibition of β-catenin activity in TgCD97 mice restored the inflammatory LPS-response partially. This identifies CD97 as a previously unrecognized context-dependent regulator of developmental endotoxin sensitivity in the immature small intestine.

### CD97 overexpression reduced the susceptibility of immature small intestinal tissues and IECs

Postnatal stages of mice display, similar to pre-term infants, an excessive intestinal inflammatory response upon LPS challenge due to the degree of intestinal immaturity. This immaturity is reflected for example by the absence of specific cells, reduced expression of protective mucins and elevated expression of TLR-4 [21]. Upon maturation, cellular mechanisms achieve homeostasis, promoting tolerance to bacterial compounds and other commensal organisms’ epitopes [22].

Within these immature stages of murine development, we observed a high degree of LPS-induced pro-inflammatory gene expression in WT IECs which was attenuated in TgCD97 IECs. Importantly, this effect was restricted to early developmental stages (P7-P28), whereas adult IECs were largely unresponsive to LPS, indicating a regulatory role of developmental endotoxin sensitivity, a feature not previously attributed to this receptor. When we analyzed KO CD97 IECs with respect to their inflammatory response we obtained similar results to WT IECs. This suggests that CD97 is dispensable for mounting a robust and balanced pro-inflammatory response under physiological conditions but that its overexpression confers a protective phenotype specifically in immature, LPS-responsive tissue. While the absence of endogenous CD97 did not increase enterocyte susceptibility to LPS, its overexpression affected the inflammatory response only in LPS-responsive age groups (P7, P14, and P28). This suggests that CD97 itself is not embedded in the endogenous LPS/TLR-4 axis in IECs but rather mediates its effect indirectly.

Next, we further investigated previously identified interaction partners linking CD97 with the TLR4/NF-κB axis in other cell types and experimental models.

### CD97-stabilized β-catenin influences inflammatory responses of IECs.

Initially, the transgenic mouse model of CD97 overexpression was established to study the function of CD97 in colorectal carcinogenesis. Upon azoxymethane DSS-induced colitis-associated tumorigenesis it was observed that TgCD97 mice exhibited reduced histological signs of DSS-colitis and inflammatory cytokine secretion [14]. In a series of different experiments, the authors showed that the underlying mechanisms was attributed to an increased structural integrity of adherens junctions by stabilizing junctional β-catenin. This locoregional accumulation of β-catenin was postulated as primary conveyor to the inflammation-attenuating effect of CD97 in these transgenic tissues.

Under normal conditions, β-catenin is marked for proteasomal degradation by its negative regulator glycogen synthase kinase 3β (GSK-3β). Together with tumor suppressors like axin and adenomatous polyposis coli (APC), GSK-3β in the destruction complex continuously phosphorylates β-catenin, maintaining a homeostatic balance by limiting the endogenous pool of available β-catenin [23]. In experiments on the transgenic mouse line TgCD97 used in our study, the stabilization of β-catenin in enterocytes overexpressing CD97 has been shown to be conveyed via the inactivation of GSK-3β [14].

We confirmed an accumulation of β-catenin in CD97 overexpressing IECs in both cytosol as well as the nuclear compartment. Since mRNA levels of β-catenin were similar in WT and TgCD97 IECs, the stabilization of β-catenin protein was proven to occur post-translationally, influencing the balance between protein synthesis and degradation.

In the context of intestinal inflammation, β-catenin is downregulated in epithelial cells of the colon during bacteria-associated inflammation [24]. Mice infected with *Salmonella typhimurium* via oral gavage displayed elevated levels of phosphorylated β-catenin together with an increased activity of GSK-3β upon infection.

A comparable condition in the immature human intestine, likely driven by gut microbiota dysbiosis and dysregulated tissue response, is necrotizing enterocolitis. NEC is an acute inflammatory disease of mostly the small intestine, with high mortality, primarily affecting preterm infants within a few weeks after birth [25]. Excessive inflammation, triggered by bacterial signaling and facilitated by a dysregulated microbiota, leads to epithelial barrier breakdown and bacterial translocation into tissues and the bloodstream—hallmarks of this gastrointestinal emergency. Although the underlying mechanisms are still subject to clinical and molecular investigation, the TLR-4/NF-κB axis in intestinal epithelial cells has been identified as playing a critical role [6,7]. In line with this, Sodhi and colleagues could show that necrotizing enterocolitis is associated with reduced β-catenin expression in enterocytes in mice and humans [20]. In addition, blockade of enterocyte TLR-4 signaling in mice undergoing experimental NEC restored β-catenin expression, underscoring a link between TLR-4 and β-catenin signaling.

Growing evidence suggests a crosstalk between β-catenin and NF-κB signaling in the context of inflammatory conditions. However, this crosstalk seems to be tissue or cell dependent as both positive and negative cross-regulation are documented [26]. In colonic IECs, β-catenin physically interacts with NF-κB preventing its activation and translocation [24,27]. Inflammatory stimuli with consecutive GSK-3β activation disrupt the complex between β-catenin and NF-κB, allowing p65 to translocate to the nucleus and induce pro-inflammatory gene expression [26]. Similar results could be obtained in an osteoblast-like cell line: Stabilization of β-catenin either by using a commercial inhibitor or siRNA directed against GSK-3β decreased LPS-induced pro-inflammatory gene expression [28].

In our study, wild type IECs displayed a rapid activation of NF-κB by LPS, reflected by an increase in the phosphorylation status of p65 after 15 minutes with subsequent cytokine synthesis even after 180 min. In parallel, β-catenin was slightly downregulated in early timepoints of this inflammatory state. In IECs overexpressing CD97 with accumulated β-catenin in the nucleus and cytosol, the phosphorylation of p65 was suppressed, together with the attenuated pro-inflammatory gene expression data obtained before.

This indicates that interaction between CD97 overexpression-induced stabilization of β-catenin and suppressed p65 activation station following LPS/TLR4 signaling in IECs, although we could not confirm or exclude a direct physical interaction in this study. By linking CD97 to β-catenin/NF-κB crosstalk in primary intestinal epithelial cells, our study provides a novel mechanistic framework that extends beyond previous observations in colonic inflammation and highlights a previously unrecognized axis in the immature small intestine.

### iCRT3 inhibits β-catenin activity and restores endotoxin response phenotype in TgCD97 IECs

To test if β-catenin is the main conveyor of the observed TgCD97 inflammatory phenotype, we directly targeted β-catenin activity using the cell-permeable selective Wnt pathway inhibitor iCRT3. iCRT3 inhibits the β-catenin/T-cell-factor (TCF) interaction by binding to β-catenin, thereby preventing TCF/LEF (lymphoid enhancer-binding factor)-dependent gene expression [29]. This led to an increased LPS susceptibility as seen in an increase in Tnf-α, Cxcl1 and Il-1β expression upon challenge compared to control cells. We cannot predict the exact mechanism by which iCRT3 renders TgCD97 IECs sensitive towards cells, as this might involve mechanisms on the transcriptional level downstream. Target gene expression of the TCF/LEF-β-catenin complex was not analyzed. Even though iCRT3 has been identified as one of the most specific small molecule inhibitors of β-catenin, related pathways like Notch and Hippo still are affected [30]. However, our data provides causal evidence that β-catenin stabilization is required for the CD97-mediated attenuation of inflammatory signaling acting as a functional mediator of CD97-depenedent endotoxin tolerance in IECS. A more favorable approach to interfering with β-catenin activity could be its downregulation using Proteolysis Targeting Chimeras (PROTACs) [31]. These heterobifunctional molecules, composed of two domains—one targeting the protein and the other binding to the E3 ligase—induce intracellular, protein-specific degradation by bringing the target protein into proximity with the E3 ligase, leading to its ubiquitination and subsequent proteasomal degradation [32]. PROTACs could mimic functional GSK-3β in TgCD97 IECs, targeting β-catenin protein directly for proteasomal degradation. On the other hand, this class of molecules might serve to imitate the effect of CD97 overexpression by decreasing activity of the β-catenin destruction complex, by targeting GSK-3β, axin or APC in the context of acute inflammation*.* Applicability and consequences of interfering with these central and conserved cellular pathways in mammalian enterocytes remains to be elucidated.

### Limitations

We did not further characterize protein expression regulation of β-catenin and GSK-3β activation status in TgCD97 or CD97 KO-derived IECs, as this has been previously documented for these specific mouse lines [14]. Additionally, we did not examine LPS-mediated GSK-3β regulation in wild-type IECs across different stages of gastrointestinal development. It is possible that early-stage IECs may have distinct regulatory mechanisms involving the β-catenin/GSK-3β axis following LPS stimulation, suggesting that developmental regulation may contribute to increasing LPS tolerance over time. We were unable to visualize total p65 in our Western blot data for comparison to the phosphorylated form.

### How can the current findings be used to further advance research into acute neonatal intestinal inflammation?

The current findings can advance research on acute neonatal intestinal inflammation: By examining the susceptibility of this mouse model to established experimental NEC protocols — such as those induced by ischemia/reperfusion or fecal transplantation — insights will be gained into the protective effects of stabilized β-catenin on disease development and progression. Since GSK-3β activity is significantly upregulated in NEC, leading to β-catenin degradation, this model provides a unique advantage over postnatal pharmacological or chemical β-catenin stabilization, as the elevation of β-catenin in this transgenic line is tissue-specific, thereby minimizing off-target systemic effects and maintaining homeostasis within the tissues. Confirmation of direct protein-protein interaction of β-catenin and the NF-κB complex or its regulators as described in other cells is pending.

### Summary

In summary, the overexpression of CD97 in small intestinal IECs reduces neonatal endotoxin susceptibility and NF-κB signaling and is associated with post-translational β-catenin stabilization. Our results suggest that this molecular setup provides resistance to endotoxin challenge by interfering with LPS-regulated pathways. This mechanism may provide a protective effect by suppressing p65-dependent pro-inflammatory gene induction, especially at immature stages of intestinal development when postnatally acquired negative regulators of TLR4/NF-κB signaling are not fully developed.

Our study demonstrates that CD97 overexpression in intestinal epithelial cells attenuates LPS-induced pro-inflammatory responses during early stages of intestinal development by stabilizing β-catenin and suppressing NF-κB activation. These findings highlight CD97’s potential as a protective modulator in neonatal intestinal inflammation, offering insights into therapeutic strategies for conditions like NEC. Further research is needed to explore its translational applicability and underlying mechanisms in human systems.

## Supporting information

S1 File PCR Dataset. (XLSX)

S2 File Western Blot quantitative data densiometrie. (XLSX)

S3 File Raw images for Western blot data. (PDF)
