# Supplementary material for: Overexpression of CD97 in intestinal epithelial cells attenuates LPS-induced pro-inflammatory cytokine induction via stabilization of β-catenin early in life
Source: PLoS One. 2026 Jul 30;21(7):e0354507. doi: 10.1371/journal.pone.0354507 (PMC13423036; doi:10.1371/journal.pone.0354507)
Supplement: S3 File — (PDF) [file pone.0354507.s003.pdf]

S1 Raw images for Western blot data

Original unadjusted and uncropped Western blot images underlying the blot data presented in Figure 4A, Figure 4D, and Figure 4F. The original TIFF image files are provided separately and have not been cropped or adjusted. The images reproduced in this PDF are lossless display copies generated solely for organization and figure-to-raw-data assignment.

Figure 4A

p-p65 (S536)

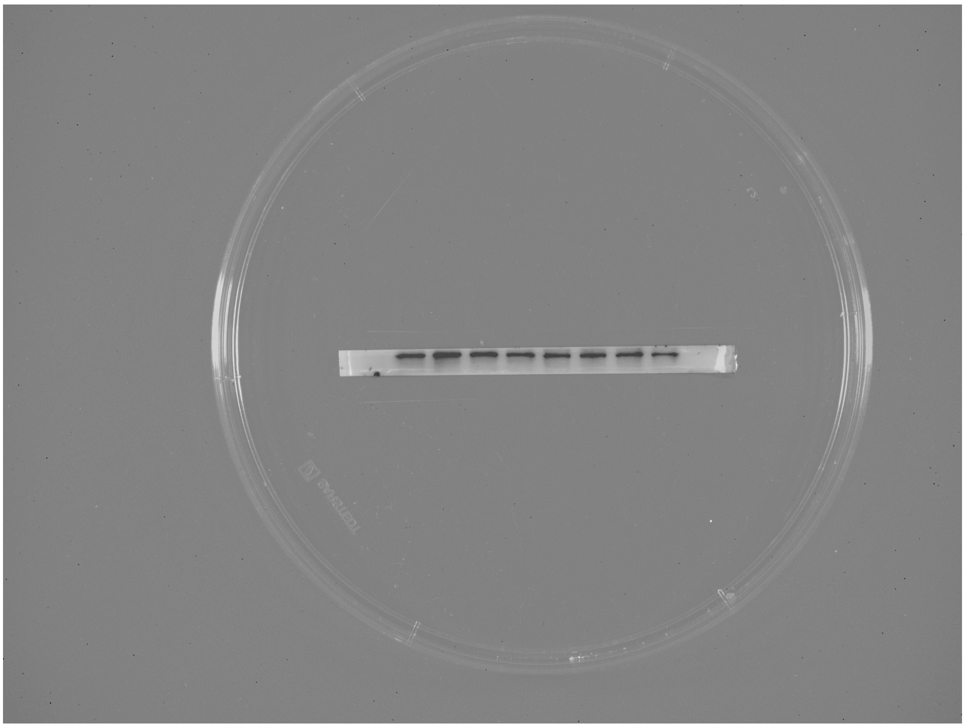

β-actin

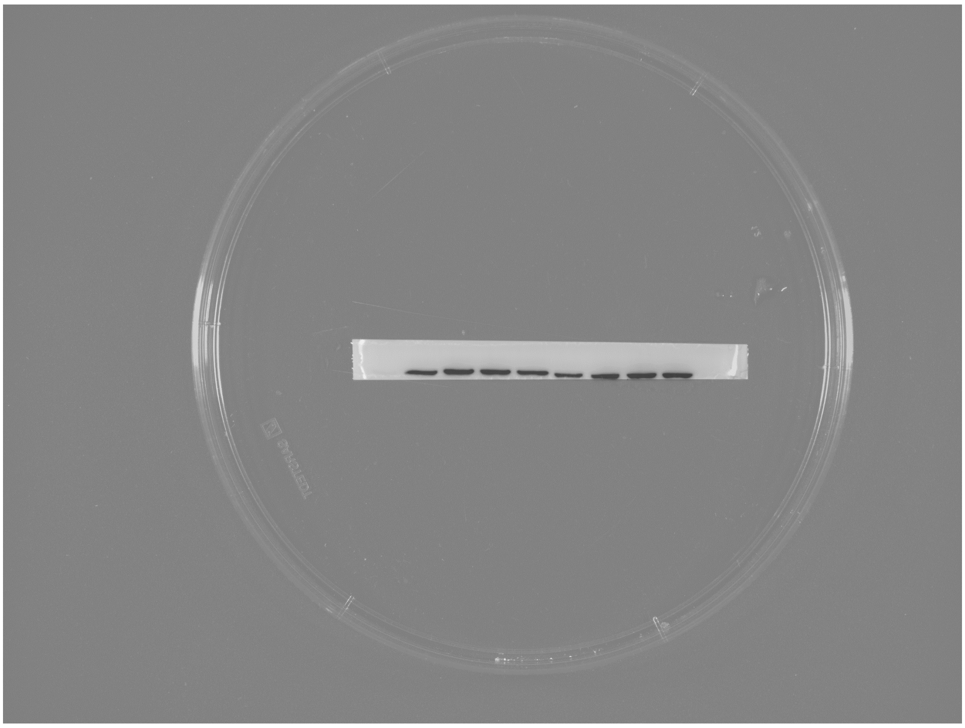

Original unadjusted and uncropped Western blot images underlying the data presented in Figure 4A.

Figure 4D

β-catenin

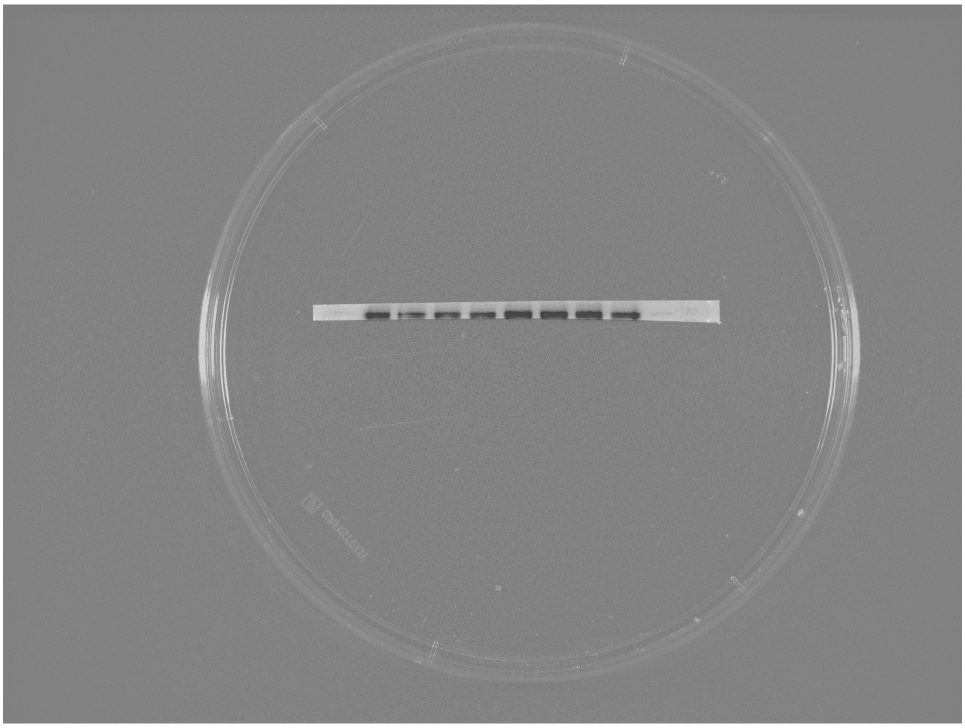

β-actin

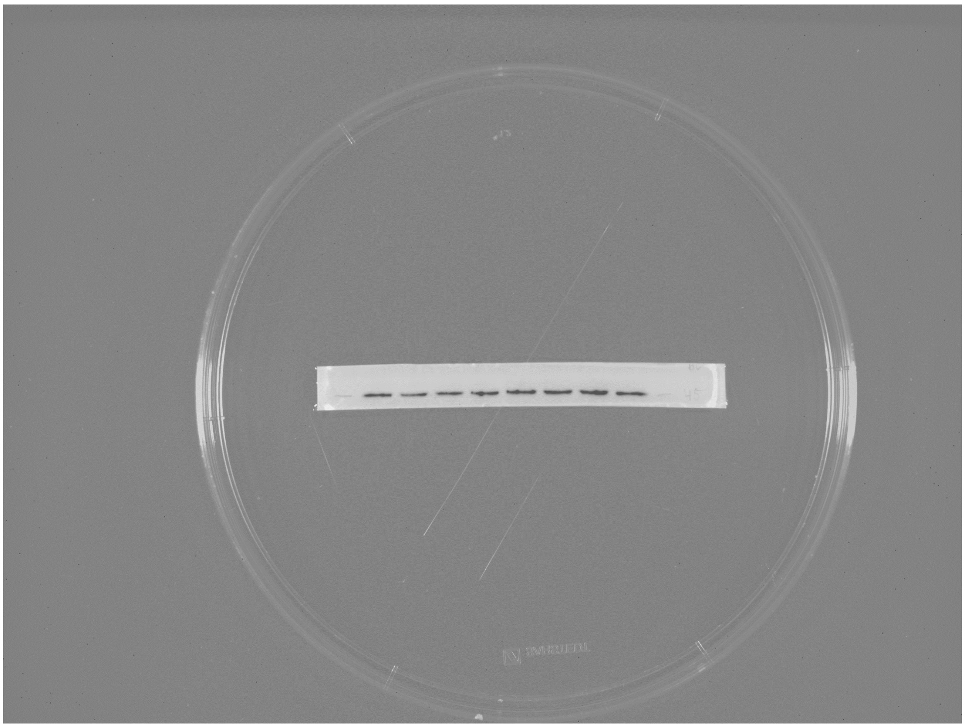

Original unadjusted and uncropped Western blot images underlying the data presented in Figure 4D.

Figure 4F

β-catenin

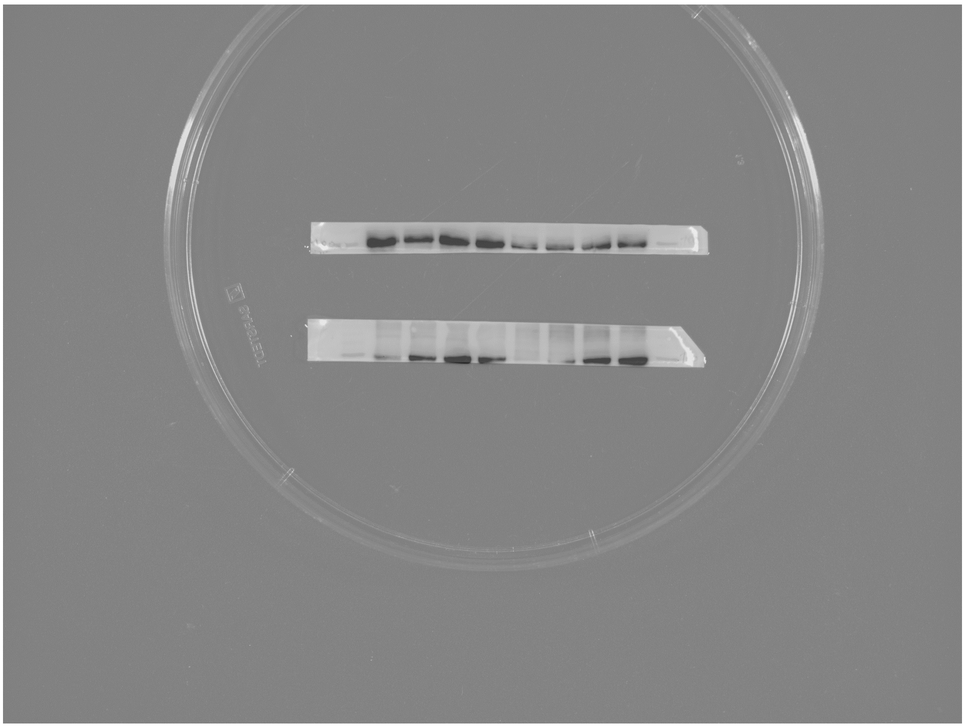

GAPDH

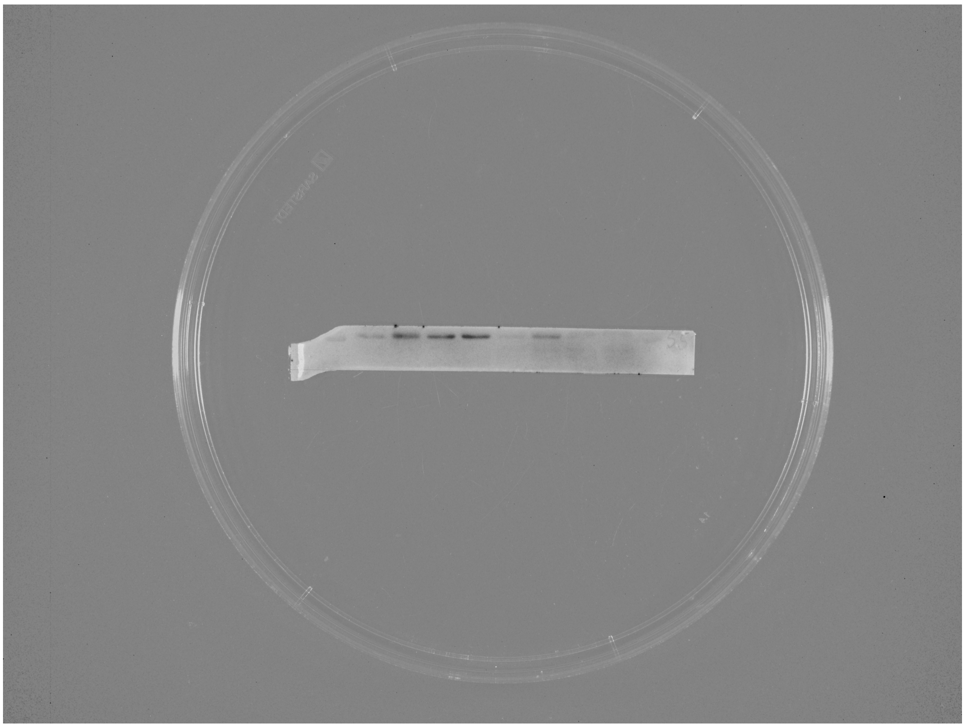

H3

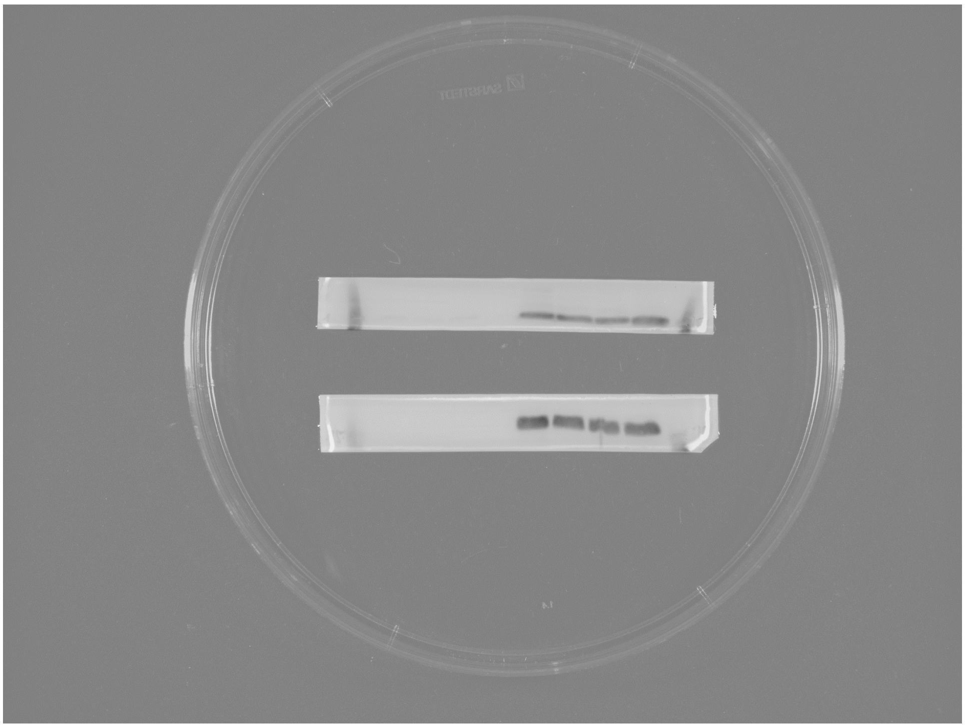

Original unadjusted and uncropped Western blot images underlying the data presented in Figure 4F.
