# Supplementary material for: Overexpression of CD97 in intestinal epithelial cells attenuates LPS-induced pro-inflammatory cytokine induction via stabilization of β-catenin early in life
Source: PLoS One. 2026 Jul 30;21(7):e0354507. doi: 10.1371/journal.pone.0354507 (PMC13423036; doi:10.1371/journal.pone.0354507)
Supplement: Attachment — Submitted filename: Response to Reviewers.docx [file pone.0354507.s005.docx]

Niklas Dressler

Department of Pediatric Surgery

University Hospital of Leipzig

Liebigstraße 20a, 04103 Leipzig, Germany

[niklas.dressler@gmail.com](mailto:Niklas.Dressler@medizin.uni-leipzig.de)

PLOS One Editoral Office

1875 Mission Street

Suite 103#188

San Francisco, CA94103

USA

**Rebuttal letter**

**For the Revision of the Manuscript “*Overexpression of CD97 in intestinal epithelial cells attenuates LPS-induced pro-inflammatory cytokine induction via stabilization of β-catenin early in life”* after Peer Review.**

Dear Editors and Reviewers,

We would like to express our sincere gratitude for the constructive feedback on our manuscript titled “Overexpression of CD97 in intestinal epithelial cells attenuates LPS-induced pro-inflammatory cytokine induction via stabilization of β-catenin early in life”. The comments and suggestions have been extremely valuable in helping us to improve the manuscript. In this letter, we address the points raised from the reviewers. We believe the revision we made will clarify the concerns raised and enhance the quality of the manuscript. We hope the revised manuscript meets your expectations for the submission to PLOS One.

This letter is structured as follows:

- Each point raised by the reviewers is addressed consecutively.
- The reviewer´s original comments are written in black.
- The response from our side to these comments are written in blue.
- Changes made to the manuscripts are printed in *blue italic*.
- Changes within the manuscript can also be tracked from the comments by an indication of the Line number which revers to the “Manuscript with Track Changes” file.

Below, we provide detailed responses to each reviewer´s comments:

**Reviewer 1**

This manuscript demonstrates that CD97 overexpression in small intestinal epithelial cells decreases neonatal susceptibility to endotoxin and dampens NF-κB signalling through post-translational stabilization of β-catenin. By interfering with LPS-regulated pathways, this mechanism suppresses p65-dependent pro-inflammatory gene expression. This protective effect is particularly important during early stages of intestinal development, when postnatally acquired negative regulators of TLR4/NF-κB signalling are not fully developed. Overall, the findings indicate that CD97 functions as a protective modulator of intestinal inflammation by stabilizing β-catenin and limiting NF-κB–mediated inflammatory responses. These insights highlight the potential relevance of CD97-mediated signaling in protecting the immature intestine and suggest possible therapeutic implications for neonatal inflammatory disorders such as necrotizing enterocolitis. The experiments are well designed and executed, and addressing a few concerns would further strengthen the study and enhance its impact.

Comments:

Major

Comment 1:

Western blots are shown in several figures; however, densitometric quantification and statistical analysis are lacking. Including these analyses would strengthen the interpretation and significance of the protein expression data.

Response 1:

We thank reviewer 1 for this suggestion and added quantitative densiometric measurements as figures as well as statements within the text. In detail we added a statement within the material and methods section about the quantitative densiometric measurement as follows: “*Densiometric quantification of western blot data was performed with Image J (Image J 1.54r).”* (Line 242).

Furthermore, we quantified the degree of phosphorylation p65 upon LPS treatment as depicted now in Fig. 4B. We referred to this figure within the text: *“Densiometric quantification of phosphorylated p65 revealed a significant difference between WT and TgCD97 after 15 minutes of LPS treatment (Fig. 4B). The overall level of phosphorylated p65 in TgCD97 IECs remained stable throughout the LPS challenge. WT p-p65 levels started to recover back to unstimulated levels at three hours after endotoxin challenge.”* (Line 379). Accordingly, we expanded the figure legend: *“(B) Densiometric quantification of phosphorylated p65 (S536) related to β-actin over the course of time. Values are depicted as Fold change related to t=0 (n=3).”* (Line 414).

Similar, densiometric quantification of β-catenin was performed and added: *“**Densiometric quantification of β-catenin revealed a significant difference between WT and TgCD97 at 15 minutes of LPS treatment (Fig. 4E).”* (Line 401). The figure legend was expanded with: “(D) *Densiometric quantification of β-catenin related to β-actin over the course of time. Values are depicted as Fold change related to t=0 (n=3).”* (Line 19).

The amount of cytosolic and nuclear β-catenin was also determined and incorporated within the text: *“Densiometric quantification of cytosolic and nuclear β-catenin levels revealed a significant difference between WT and TgCD97 derived IECs (Fig. 4G).”* (Line 401) as well as within the figure legends: *“(G) Densiometric quantification of cytosolic β-catenin between WT and TgCD97 IECs. Cytosolic β-catenin levels were related to GAPDH (n=3). (H) Densiometric quantification of nuclear β-catenin between WT and TgCD97 IECs. Nuclear β-catenin was related to H3 (n=3).”* (Line 425)

Comment 2:

In Figure 5C, the GAPDH blots appear blurry and are difficult to interpret. The authors should provide clearer blots for proper evaluation. Alternatively, β-actin could be used as a cytoplasmic loading control for normalization

Response 2:

We thank reviewer 1 for this suggesting and followed his advice by providing a clearer GAPDH signal. At this point we would like to mention that due to figure re-organisation the order of the graph has changed and the GAPDH bot can now be found as Fig. 4F.

Comment 3:

Also, in Figure 5C, two wild-type and three TgCD samples are mentioned in the legends; however, only one wild-type expression band appears to be shown in the blot. The authors should clarify this discrepancy and provide the corresponding data for all the mentioned samples.

Response 3:

We sincerely apologize for this discrepancy. Within the Western Blot analysis we only used one P14 WT mouse as representative. We change this within the figure legends as stated: *“(F) Representative western blot for the distribution of β-catenin from one P14 WT and three P14 TgCD97 (TgCD97 #1, #2, #3) within the cytosolic and nuclear fraction.”* (Line 423).

Comment 4:

The manuscript would benefit from including concise descriptions of the generation of CD97 overexpressing and knockout mice in the Methods section, to enhance clarity and reproducibility

Response 4:

We thank reviewer 1 for these suggestions and added a detailed description of the generation of the CD97 overexpression and knockout mice in the Methods sections. In detail we added: *„ In brief, for the generation of TgCD97 mice, a 9kb regulatory region of the mouse villin gene was place upstream of a CD97 cDNA to generate a villin-CD97 expression construct. Pronuclear microinjection of the purified expression construct into fertilized oocytes of C57BL/6J mice was performed to generate TgCD97 mice. Founders were mated to wild-type mice.”* (Line 159). With respect to the generation of CD97 knout-out mice we added: “*CD97 knout-out mice were generated using Cre/loxP-mediated DNA recombination. The targeting construct consisted of a loxP-PGKneo-loxP cassette which was inserted upstream of exon 2 and a third loxP site was placed downstream of exon 5 of the murine CD97 gene spanning intron 1 to exon 12. The linearized targeting construct was electroporated into 129/Ola-derived embryonic stem (ES) cells. Selection with G418 was performed for 8 days and G418-resistant ES cell clones were used to PCR based verification of homologous recombination. Subsequently clone IIH10 was microinjected into C57BL/6J-derived blastocytes to generate chimeric offspring which were mated with C57BL/6J mice. Mice positive for germline transmission were mated with homozygous EIIaCre mice expressing Cre recombinase during embryonic development. CD97 deficient mice were backcrossed on a C57BL/6J background.”* (Line 163).

Comment 5:

In Figure 3, please include a significance bar comparing treated and untreated CD97 knockout samples to better illustrate their similarity to the wild-type condition. This addition would strengthen the interpretation of whether the treatment mimics the wild-type phenotype

Response 5

We thank Reviewer 1 for highlighting these opportunities for improvement and have added the requested significance bars. As two-way ANOVA accounts for variability across all groups in pairwise comparisons, the differences between wild-type controls and LPS-stimulated IECs did not reach statistical significance. These results further support the similarity between WT and CD97 KO datasets.

Comment 6:

The use of same TNFα and IL-1β values for wild-type mice in both the CD97 overexpression and CD97 knockout experiments is concerning and raises questions regarding the independence of the datasets. The authors must clarify whether the same wild-type control data were used for both experiments and provide a clear justification, as the use of identical control data across separate experiments is generally not acceptable

- Fig 1B and 3A
- Fig 1C and 3B
- Fig 1F and 3C
- Fig 1G and 3D

Response 6:

We thank reviewer 1 for raising the important point and fully agree that transparency regarding data origin is essential.

We would like to clarify that the wild-type (WT) control data used in this study were generated as part of the same experimental series and have been partially reported in a previous publication. The reuse of these WT datasets was intentional, as they serve as a shared baseline control for multiple genetically modified mouse lines analysed under identical experimental conditions (age, tissue, isolation, stimulation protocol and reagents). We chose this approach to ensure direct comparability between CD97 overexpression and CD97 knockout models, while minimizing experimental variability that could arise from independently generated control groups. Importantly, the WT database are not presented as novel datasets in this manuscript. Our main focus and novelty presented in this study lies not in the WT baseline itself, but in the comparative analysis of the CD97 overexpression and knockout as well as the identification of β-catenin mediated regulation of inflammatory signaling in intestinal epithelial cells. To address this concern, we have revised the manuscript and explicitly stated in the methods section that WT control were reused and previously published.

In detail we made several remarks:

*“WT control database used in this study were generated as part of the same experimental series and have been partially reported as previously. These data were reused to ensure direct comparability between experimental groups under the same experimental conditions.” (*Line 205 - Methods section):

Additionally, we have clearly indicated in the figure legends which data have been reported before.

“*WT control data have been partially published previously and are shown here for direct comparison.”* (Line 288 Figure 1 - Legend):

“*WT control data shown in this figure are identical to those presented in Figure 1 and were derived from the same experimental dataset which have been partially published previously and are shown here for direct comparison.”* (Line 334 Figure 3 Legend):

We also provided the raw data in the supplementary material.

We believe that this transparent approach ensures scientific rigor while allowing robust comparison across experimental conditions.

We would like to emphasize that we contacted PLOS One with respect to the WT database and attached the original message from our side:

--------------- Original Message ---------------
From: Niklas Dressler [[em@editorialmanager.com](mailto:em@editorialmanager.com)]
Sent: 2/6/2026, 3:14 AM
To: [plosone@plos.org](mailto:plosone@plos.org)
Subject: Author Query to Editor PONE-D-26-04060; Disclosure of Previously Published Partial Dataset - [EMID:d20cf7804f916212]

EXTERNAL EMAIL – SECURITY NOTICE
This message originated outside of PLOS. Exercise caution when interacting with links, attachments, or requests for sensitive information.

EXTERNAL EMAIL – SECURITY NOTICE
This message originated outside of PLOS. Exercise caution when interacting with links, attachments, or requests for sensitive information.

Manuscript information:
========================
PONE-D-26-04060
Overexpression of CD97 in intestinal epithelial cells attenuates LPS-induced pro-inflammatory cytokine induction via stabilization of β-catenin early in life
PLOS One
========================
Dear Editors,

thank you very much for your time and for handling our submitted manuscript.

We would like to inform you that, contrary to our initial information during submission, a small part of the dataset has already been published in a separate article. This concerns the raw qRT-PCR data of TNF-α expression in wild-type intestinal epithelial cells with and without LPS stimulation.

These data were generated in parallel with another project investigating age-dependent inflammatory mediator patterns in intestinal epithelial cells and the coordinated response of intraepithelial lymphocytes to endotoxin stimulation. The corresponding article was published in Paediatric Research on July 21, 2025:

Murine intestinal epithelial cells and lymphocytes undergo contrasting inflammatory shifts during gastrointestinal development
<https://doi.org/10.1038/s41390-025-04262-z>

The relevant data are presented in Figure 3A of that publication and correspond to Figure 1A–D in our submitted manuscript, where the red bars represent the previously published dataset.

We would like to emphasize that the primary focus of the present manuscript is the comparative analysis between wild-type and CD97-overexpressing intestinal epithelial cells across different developmental stages. Therefore, the scientific scope, research questions, and main conclusions of the two studies differ substantially.

We sincerely apologize for this oversight and for any inconvenience it may cause. We fully appreciate the importance of transparency and would be grateful for your guidance on how to proceed.

Thank you very much for your understanding and consideration.

Kind regards,

Niklas Dressler

on behalf of the authors

Comment 7:

The novelty of the study is not well established. Multiple previous studies have already demonstrated the role of CD97 following LPS induction; therefore, the authors must clearly define the novel aspect of this work and explain how it advances current understanding beyond what is already known.

Response 7:

We thank reviewer 1 for this important comment. We agree that CD97 has previously been implicated in the regulation of inflammatory responses following LPS stimulation, particulary in macrophages and in models of colonic inflammation. However, the present study provides several novel aspects that extend current understanding in a biologically and clinically relevant manner:

- First characterization of CD97 function in immature small intestinal epithelium

Previous studies have primarily focused on immune cells (e.g. macrophages) or adult colonic tissue. To our knowledge, this is the first study investigating CD97 function specifically in primary small intestinal epithelial cells (IECs) across defined postnatal developmental stages that model human neonatal gut maturation.

- Demonstration of an age-dependent developmentally restricted effect

We show that CD07-mediated attenuation of LPS-induced inflammation is restricted to early developmental stages (P7-P28), while adult IECs are largely unresponsive. This identifies CD97 as a regulator of developmental endotoxin sensitivity, which has not been previously described.

- Tissue-specific effect in the ileum

Our data demonstrates that the anti-inflammatory effect of CD97 is restricted to ileal tissue, the primary site of necrotizing enterocolitis (NEC). This spatial specificity has been reported before and provides translational relevance to neonatal intestinal disease.

- Functional validation via pharmacological inhibition of β-catenin

By using the inhibitor iCRT3, we demonstrate that blocking β-catenin signaling restores the pro-inflammatory response in CD97-overexpressing IECs. This provides causal evidence linking β-catenin stabilization to the observed phenotype.

- Relevance to neonatal disease mechanisms (NEC)

By integrating developmental timing, ileal specificity, and β-catenin/NF-κB crosstalk, our study provided a mechanistic framework linking CD97 to endotoxin tolerance in the immature gut, which is highly relevant to NEC pathogenesis.

To address these novelties within the manuscript we made several changes within the manuscript.

- “*This effect was restricted to early developmental stages and ileal tissue, highlighting a previously unrecognized role of CD97 in age-dependent endotoxin tolerance. These findings identify a novel CD97-β-catenin-NF-κB regulatory axis in the immature small intestine* *and* their potential as targets in protecting the immature gut of neonates from inflammatory damage.” (Line 50 Abstract).
- *“While CD97 has previously been implicated in the regulation of LPS-induced inflammatory signaling in immune, its role in the immature small intestinal epithelium has not been defined.”* (Line 466 Discussion).
- *“This identifies CD97 as a previously unrecognized context-dependent regulator of developmental endotoxin sensitivity in immature intestine.”* (Line 474 Discussion).
- *“Importantly, this effect was restricted to early developmental stages (P7-P28), whereas adult IECs were largely unresponsive to LPS, indicating a regulatory role of developmental endotoxin sensitivity, a feature not previously attributed to this receptor.”* (Line 488 Discussion).
- “This suggests that CD97 is dispensable for mounting a robust and balanced pro-inflammatory response *under physiological conditions but that its overexpression confers a protective phenotype specifically in immature, LPS-responsive tissue*.” (Line 493 Discussion).
- *“By linking CD97 to β-catenin/NF-κB crosstalk in primary intestinal epithelial cells, our study provides a novel mechanistic framework that extends beyond previous observations in colonic inflammation and highlights a previously unrecognized axis in the immature small intestine.”* (Line 590 Discussion).
- *“However, our data provides causal evidence that β-catenin stabilization is required for the CD97-mediated attenuation of inflammatory signaling acting as a functional mediator of CD97-depenedent endotoxin tolerance in IECS.”* (Line 607 Discussion).

Minor

Comment 1:

Please include a comma in the first line of the Author Summary after “(NEC)” and also in the first line of the discussion section after the word “study” to improve readability. Lines 53 and 389

Response 1:

We thank reviewer 1 for these hints to produce readability and we included the suggested commas at the indicated positions.

Comment 2:

The Methods section (Immunoblot analysis) states that cytoplasmic proteins were isolated by centrifuging the supernatant at 'maximum speed' for 15 min. Please provide the exact centrifugation speed to ensure reproducibility. Line 201

Response 2:

We thank reviewer 1 for this remark and change the wording “maximum speed” to “*18000g*”. (Line 234).

Comment 3:

It is suggested to insert the word “of” between ‘context’ and ‘acute’ to improve readability. Line 527

Response 3:

We thank reviewer 1 for this suggestion and introduced the word “of” between “context” and “acute” and change the sentence to *“**On the other hand, this class of molecules might serve to imitate the effect of CD97 overexpression by decreasing activity of the β-catenin destruction complex, by targeting GSK-3β, axis or APC in the context of acute inflammation.”* (Line 616)

Comment 4:

Abbreviations should be defined where used for the first time and then consistently used in the manuscript

Response 4:

We thank Reviewer 1 for pointing out these inconsistencies in the use of abbreviations. The manuscript has been revised accordingly, and all abbreviations are now defined at first use and used consistently throughout.

**Reviewer 2**

Study by Niklas Dressler et al, entitled “Overexpression of CD97 in intestinal epithelial cells attenuates LPS-induced proinflammatory cytokine induction via stabilization of β-catenin early in life”. The authors studied the impact of IEC-specific CD97 overexpression on LPS-induced cytokine expression in isolated IECs. There are several concerns, and addressing them will strengthen the study.

Comment 1:

While TNF upregulation was significantly reduced in IEC-specific CD97 overexpressing cells, IL-1β remained unchanged. Since NEC pathogenesis involves multiple cytokines acting collectively, assessing individual cytokines provides only a partial view.

We thank reviewer 2 for this important comment and agree that NEC pathogenesis involves a complex network of interacting inflammatory mediators. In the present study, our aim was not to provide a comprehensive cytokine profiling, but to investigate the mechanistic impact of CD97 overexpression on LPS-induced signaling in immature intestinal epithelial cells. Therefore, we focused on selected NF-κB target genes as representative readouts. We observed a significant reduction inf *Tnf-α* gene expression whereas *Il-1β* was not significantly altered. This indicates that CD97 does not uniformly suppress all pro-inflammatory cytokines but rather exerts a selective regulatory effect on specific downstream targets. Importantly, this interpretation is supported by our upstream signaling data showing that LPS-induced phosphorylation of NF-κB is increased in WT IECs but absent in CD97 overexpressing IECs. This suggests that CD97 interferes with the inducible activation of NF-κB pathways in response to LPS rather than reducing basal signaling activity. The lack of uniform changes across all cytokines despite altered NF-κB activation suggests differential sensitivity of individual target genes to NF-κB signaling which is known to depends on promoter context, co-regulatory factors and activation thresholds. Furthermore, inhibition of β-catenin restored pro-inflammatory gene expression, demonstrating that CD97-mediated modulation occurs at the level of signaling dynamics rather than broad suppression of cytokine production.

In addition, we would like to emphasize that NEC represents a multifactorial disease involving complex interactions between microbial signals, hypoxia/ischemia, barrier dysfunction, and prolonged inflammatory responses over time. Established *in vivo* models of NEC typically combine multiple stressors such as hypoxia, formula feeding and microbial exposure over extended periods. In contrast, our experimental approach utilizes a simplified and controlled *in vitro* model based on short-term LPS stimulation of primary intestinal epithelial cells. This reductionist setup was intentionally chosen to isolate epithelial-intrinsic signaling mechanisms and to mechanistically dissect the interaction between CD97, β-catenin and NF-κB in response to defined inflammatory stimulus. While this approach does not recapitulate the fully complexity of NEC pathogenesis, it models a key component of the disease, namely exaggerated epithelial responses to bacterial endotoxins in the immature intestine. Thus, our findings should be interpreted as identifying a mechanistic element of epithelial inflammatory regulation that may contribute to NEC development, rather than as a comprehensive representation of the disease.

Comment 2:

Better representative blot should be provided for PPAR-γ, as the internal control shows considerable variation between the control and treatment groups. Additionally, a bar graph for densitometric analysis should be included.

Response 2:

We thank reviewer 2 for this helpful comment. We carefully re-evaluated the PPAR-γ Western blot data and performed additional experiments to improve reproducibility and loading consistency. However, we were unable to obtain sufficiently robust and reproducible results, particularly due to variability in the loading control. Therefore, we decided to remove the PPAR-γ data from the manuscript to avoid overinterpretation and to ensure the overall quality and reliability of the study. Therefore, we change the figure-heading from figure 4 to: “*CD97 overexpression suppresses NF-κB activation and is associated with post-translational stabilized β-catenin*.” (Line 408)

Comment 3:

Nuclear PPAR-γ levels should also be measured, as PPAR-γ activation is best assessed by its nuclear localization.

Response 3:

We thank reviewer 2 for this important suggestion and agree that nuclear localization represents a key indicator of PPAR-γ activation. However, due to the limitations in mentioned in Comment 2) we did not analyze nuclear localization of PPAR-γ.

Comment 4:

In Figure 5, the Y-axis label for β-catenin should be corrected for spelling.

Response 4:

We thank reviewer 2 for this clarification and checked spelling of β-catenin in all figures. Please note, that due to figure re-organization Figure 5 has become Figure 4.

Comment 5:

The statistical analysis for the figure should be re-examined. For example, in Figure 1F, data points in the blue bar appear to have minimal error, yet the results are not significant.

Response 5:

We thank Reviewer 1 for this important comment. We have re-examined the statistical analyses for all relevant figures and corrected inconsistencies in the analysis settings. Specifically, the two-way ANOVA (factors: genotype and treatment) was consistently applied across datasets, and the appropriate multiple comparisons settings were used.

These adjustments led to minor changes in significance levels in some cases. Importantly, the lack of statistical significance in Figure 1F was due to an incorrect multiple comparisons setting in the initial analysis. After correction, this comparison is now statistically significant, consistent with the observed low variability within the control.

All statistical analyses have now been recalculated, verified, and clearly annotated in the revised figures and figure legends.

Comment 6:

For the cytoplasmic fraction in Figure 5C, the GAPDH blot is of poor quality and should be replaced with clearer, more reliable data.

Response 6:

We thank reviewer 2 for this suggestion and replaced the GAPDH blot with one having a better quality. Please note that Figure 5C changed to Figure 4F.

We sincerely thank the reviewers and editors for their time, effort, and insightful feedback, which have greatly contributed to improving the quality of our manuscript. We are confident that our revisions and responses satisfactorily address the points raised, and we look forward to your feedback on the updated manuscript.

We appreciate your time and consideration in reviewing our revised manuscript.

Yours sincerely,

Jan Riedel	Niklas Dressler
