# Supplementary material for: Overexpression of CD97 in intestinal epithelial cells attenuates LPS-induced pro-inflammatory cytokine induction via stabilization of β-catenin early in life
Source: PLoS One. 2026 Jul 30;21(7):e0354507. doi: 10.1371/journal.pone.0354507 (PMC13423036; doi:10.1371/journal.pone.0354507)
Supplement: Peer review history

# Peer review: Decision Letter 0

**DOI:** 10.1371/journal.pone.0354507.r001

**Authors:** Pradeep Dudeja

2 Apr 2026

PONE-D-26-04060

Overexpression of CD97 in intestinal epithelial cells attenuates LPS-induced pro-inflammatory cytokine induction via stabilization of β-catenin early in life

PLOS One

Dear Dr. Dressler,

Thank you for submitting your manuscript to PLOS ONE. After careful consideration, we feel that it has merit but does not fully meet PLOS ONE’s publication criteria as it currently stands. Therefore, we invite you to submit a revised version of the manuscript that addresses the points raised during the review process.

The key issues include improving the quality of data and its presentation.  The authors should respond to each comments of the reviewers in a point by point manner.

Please submit your revised manuscript by May 17 2026 11:59PM. If you will need more time than this to complete your revisions, please reply to this message or contact the journal office at plosone@plos.org. When you're ready to submit your revision, log on to https://www.editorialmanager.com/pone/ and select the 'Submissions Needing Revision' folder to locate your manuscript file.

Please include the following items when submitting your revised manuscript:

- A letter that responds to each point raised by the academic editor and reviewer(s). You should upload this letter as a separate file labeled 'Response to Reviewers'.
- A marked-up copy of your manuscript that highlights changes made to the original version. You should upload this as a separate file labeled 'Revised Manuscript with Track Changes'.
- An unmarked version of your revised paper without tracked changes. You should upload this as a separate file labeled 'Manuscript'.

If you would like to make changes to your financial disclosure, please include your updated statement in your cover letter. Guidelines for resubmitting your figure files are available below the reviewer comments at the end of this letter.

If applicable, we recommend that you deposit your laboratory protocols in protocols.io to enhance the reproducibility of your results. Protocols.io assigns your protocol its own identifier (DOI) so that it can be cited independently in the future. For instructions see: https://journals.plos.org/plosone/s/submission-guidelines#loc-laboratory-protocols. Additionally, PLOS ONE offers an option for publishing peer-reviewed Lab Protocol articles, which describe protocols hosted on protocols.io. Read more information on sharing protocols at https://plos.org/protocols?utm_medium=editorial-email&utm_source=authorletters&utm_campaign=protocols.

As the corresponding author, your ORCID iD is verified in the submission system and will appear in the published article. PLOS supports the use of ORCID, and we encourage all coauthors to register for an ORCID iD and use it as well. Please encourage your coauthors to verify their ORCID iD within the submission system before final acceptance, as unverified ORCID iDs will not appear in the published article. *Only*  the individual author can complete the verification step; PLOS staff *cannot*  verify ORCID iDs on behalf of authors.

We look forward to receiving your revised manuscript.

Kind regards,

Pradeep Dudeja

Academic Editor

PLOS One

**Journal Requirements:**

When submitting your revision, we need you to address these additional requirements.

1. Please ensure that your manuscript meets PLOS ONE's style requirements, including those for file naming. The PLOS ONE style templates can be found at

https://journals.plos.org/plosone/s/file?id=wjVg/PLOSOne_formatting_sample_main_body.pdf and

https://journals.plos.org/plosone/s/file?id=ba62/PLOSOne_formatting_sample_title_authors_affiliations.pdf

2. To comply with PLOS One submissions requirements, in your Methods section, please provide additional information regarding the experiments involving animals and ensure you have included details on (1) methods of sacrifice, (2) methods of anesthesia and/or analgesia, and (3) efforts to alleviate suffering.

3. PLOS ONE now requires that authors provide the original uncropped and unadjusted images underlying all blot or gel results reported in a submission’s figures or Supporting Information files. This policy and the journal’s other requirements for blot/gel reporting and figure preparation are described in detail at https://journals.plos.org/plosone/s/figures#loc-blot-and-gel-reporting-requirements and https://journals.plos.org/plosone/s/figures#loc-preparing-figures-from-image-files. When you submit your revised manuscript, please ensure that your figures adhere fully to these guidelines and provide the original underlying images for all blot or gel data reported in your submission. See the following link for instructions on providing the original image data: https://journals.plos.org/plosone/s/figures#loc-original-images-for-blots-and-gels.

In your cover letter, please note whether your blot/gel image data are in Supporting Information or posted at a public data repository, provide the repository URL if relevant, and provide specific details as to which raw blot/gel images, if any, are not available. Email us at plosone@plos.org if you have any questions.

4. Please include captions for your Supporting Information files at the end of your manuscript, and update any in-text citations to match accordingly. Please see our Supporting Information guidelines for more information: http://journals.plos.org/plosone/s/supporting-information.

5. We note that there is identifying data in the Supporting Information file < S1_Supplementary_Data_qPCR.xlsx >. Due to the inclusion of these potentially identifying data, we have removed this file from your file inventory. Prior to sharing human research participant data, authors should consult with an ethics committee to ensure data are shared in accordance with participant consent and all applicable local laws.

Data sharing should never compromise participant privacy. It is therefore not appropriate to publicly share personally identifiable data on human research participants. The following are examples of data that should not be shared:

-Name, initials, physical address

-Ages more specific than whole numbers

-Internet protocol (IP) address

-Specific dates (birth dates, death dates, examination dates, etc.)

-Contact information such as phone number or email address

-Location data

-ID numbers that seem specific (long numbers, include initials, titled “Hospital ID”) rather than random (small numbers in numerical order)

Data that are not directly identifying may also be inappropriate to share, as in combination they can become identifying. For example, data collected from a small group of participants, vulnerable populations, or private groups should not be shared if they involve indirect identifiers (such as sex, ethnicity, location, etc.) that may risk the identification of study participants.

Additional guidance on preparing raw data for publication can be found in our Data Policy (https://journals.plos.org/plosone/s/data-availability#loc-human-research-participant-data-and-other-sensitive-data) and in the following article: http://www.bmj.com/content/340/bmj.c181.long.

Please remove or anonymize all personal information ( ID numbers that seem specific), ensure that the data shared are in accordance with participant consent, and re-upload a fully anonymized data set. Please note that spreadsheet columns with personal information must be removed and not hidden as all hidden columns will appear in the published file.

6. If the reviewer comments include a recommendation to cite specific previously published works, please review and evaluate these publications to determine whether they are relevant and should be cited. There is no requirement to cite these works unless the editor has indicated otherwise.

[Note: HTML markup is below. Please do not edit.]

Reviewers' comments:

Reviewer's Responses to Questions

**Comments to the Author**

1. Is the manuscript technically sound, and do the data support the conclusions?

The manuscript must describe a technically sound piece of scientific research with data that supports the conclusions. Experiments must have been conducted rigorously, with appropriate controls, replication, and sample sizes. The conclusions must be drawn appropriately based on the data presented.

Reviewer #1: Partly

Reviewer #2: Yes

**********

2. Has the statistical analysis been performed appropriately and rigorously?

Reviewer #1: Yes

Reviewer #2: I Don't Know

**********

3. Have the authors made all data underlying the findings in their manuscript fully available?

The PLOS Data policy requires authors to make all data underlying the findings described in their manuscript fully available without restriction, with rare exception (please refer to the Data Availability Statement in the manuscript PDF file). The data should be provided as part of the manuscript or its supporting information, or deposited to a public repository. For example, in addition to summary statistics, the data points behind means, medians and variance measures should be available. If there are restrictions on publicly sharing data—e.g. participant privacy or use of data from a third party—those must be specified.

Reviewer #1: Yes

Reviewer #2: Yes

**********

4. Is the manuscript presented in an intelligible fashion and written in standard English?

PLOS ONE does not copyedit accepted manuscripts, so the language in submitted articles must be clear, correct, and unambiguous. Any typographical or grammatical errors should be corrected at revision, so please note any specific errors here.

Reviewer #1: Yes

Reviewer #2: Yes

**********

5. Review Comments to the Author

Please use the space provided to explain your answers to the questions above. You may also include additional comments for the author, including concerns about dual publication, research ethics, or publication ethics. (Please upload your review as an attachment if it exceeds 20,000 characters)

Reviewer #1: This manuscript demonstrates that CD97 overexpression in small intestinal epithelial cells decreases neonatal susceptibility to endotoxin and dampens NF-κB signalling through post-translational stabilization of β-catenin. By interfering with LPS-regulated pathways, this mechanism suppresses p65-dependent pro-inflammatory gene expression. This protective effect is particularly important during early stages of intestinal development, when postnatally acquired negative regulators of TLR4/NF-κB signalling are not fully developed. Overall, the findings indicate that CD97 functions as a protective modulator of intestinal inflammation by stabilizing β-catenin and limiting NF-κB–mediated inflammatory responses. These insights highlight the potential relevance of CD97-mediated signaling in protecting the immature intestine and suggest possible therapeutic implications for neonatal inflammatory disorders such as necrotizing enterocolitis. The experiments are well designed and executed, and addressing a few concerns would further strengthen the study and enhance its impact.

Comments:

Major

• Western blots are shown in several figures; however, densitometric quantification and statistical analysis are lacking. Including these analyses would strengthen the interpretation and significance of the protein expression data

• In Figure 5C, the GAPDH blots appear blurry and are difficult to interpret. The authors should provide clearer blots for proper evaluation. Alternatively, β-actin could be used as a cytoplasmic loading control for normalization

• Also, in Figure 5C, two wild-type and three TgCD samples are mentioned in the legends; however, only one wild-type expression band appears to be shown in the blot. The authors should clarify this discrepancy and provide the corresponding data for all the mentioned samples.

• The manuscript would benefit from including concise descriptions of the generation of CD97 overexpressing and knockout mice in the Methods section, to enhance clarity and reproducibility

• In Figure 3, please include a significance bar comparing treated and untreated CD97 knockout samples to better illustrate their similarity to the wild-type condition. This addition would strengthen the interpretation of whether the treatment mimics the wild-type phenotype

• The use of same TNFα and IL-1β values for wild-type mice in both the CD97 overexpression and CD97 knockout experiments is concerning and raises questions regarding the independence of the datasets. The authors must clarify whether the same wild-type control data were used for both experiments and provide a clear justification, as the use of identical control data across separate experiments is generally not acceptable

- Fig 1B and 3A

- Fig 1C and 3B

- Fig 1F and 3C

- Fig 1G and 3D

• The novelty of the study is not well established. Multiple previous studies have already demonstrated the role of CD97 following LPS induction; therefore, the authors must clearly define the novel aspect of this work and explain how it advances current understanding beyond what is already known

Minor

• Please include a comma in the first line of the Author Summary after “(NEC)” and also in the first line of the discussion section after the word “study” to improve readability. Lines 53 and 389

• The Methods section (Immunoblot analysis) states that cytoplasmic proteins were isolated by centrifuging the supernatant at 'maximum speed' for 15 min. Please provide the exact centrifugation speed to ensure reproducibility. Line 201

• It is suggested to insert the word “of” between ‘context’ and ‘acute’ to improve readability. Line 527

• Abbreviations should be defined where used for the first time and then consistently used in the manuscript

Reviewer #2: Study by Niklas Dressler et al, entitled “Overexpression of CD97 in intestinal epithelial cells attenuates LPS-induced proinflammatory cytokine induction via stabilization of β-catenin early in life”. The authors studied the impact of IEC-specific CD97 overexpression on LPS-induced cytokine expression in isolated IECs. There are several concerns, and addressing them will strengthen the study.

1) While TNF upregulation was significantly reduced in IEC-specific CD97 overexpressing cells, IL-1β remained unchanged. Since NEC pathogenesis involves multiple cytokines acting collectively, assessing individual cytokines provides only a partial view.

2) Better representative blot should be provided for PPAR-γ, as the internal control shows considerable variation between the control and treatment groups. Additionally, a bar graph for densitometric analysis should be included.

3) Nuclear PPAR-γ levels should also be measured, as PPAR-γ activation is best assessed by its nuclear localization.

4) In Figure 5, the Y-axis label for β-catenin should be corrected for spelling.

5) The statistical analysis for the figure should be re-examined. For example, in Figure 1F, data points in the blue bar appear to have minimal error, yet the results are not significant.

6) For the cytoplasmic fraction in Figure 5C, the GAPDH blot is of poor quality and should be replaced with clearer, more reliable data.

**********

6. PLOS authors have the option to publish the peer review history of their article (what does this mean?). If published, this will include your full peer review and any attached files.

If you choose “no”, your identity will remain anonymous but your review may still be made public.

**Do you want your identity to be public for this peer review?**  For information about this choice, including consent withdrawal, please see our Privacy Policy.

Reviewer #1: **Yes:**  Nazim Husain

Reviewer #2: No

**********

[NOTE: If reviewer comments were submitted as an attachment file, they will be attached to this email and accessible via the submission site. Please log into your account, locate the manuscript record, and check for the action link "View Attachments". If this link does not appear, there are no attachment files.]

To ensure your figures meet our technical requirements, please review our figure guidelines: https://journals.plos.org/plosone/s/figures

You may also use PLOS’s free figure tool, NAAS, to help you prepare publication quality figures: https://journals.plos.org/plosone/s/figures#loc-tools-for-figure-preparation.

NAAS will assess whether your figures meet our technical requirements by comparing each figure against our figure specifications.

# Author comment: Author response to Decision Letter 1

**DOI:** 10.1371/journal.pone.0354507.r002

2 Jun 2026

Dear Editors and Reviewers,

We would like to express our sincere gratitude for the constructive feedback on our manuscript titled “Overexpression of CD97 in intestinal epithelial cells attenuates LPS-induced pro-inflammatory cytokine induction via stabilization of β-catenin early in life”. The comments and suggestions have been extremely valuable in helping us to improve the manuscript. In this letter, we address the points raised from the reviewers. We believe the revision we made will clarify the concerns raised and enhance the quality of the manuscript. We hope the revised manuscript meets your expectations for the submission to PLOS One.

This letter is structured as follows:

• Each point raised by the reviewers is addressed consecutively.

• The reviewer´s original comments are written in black.

• The response from our side to these comments are written in blue.

• Changes made to the manuscripts are printed in blue italic.

• Changes within the manuscript can also be tracked from the comments by an indication of the Line number which revers to the “Manuscript with Track Changes” file.

Below, we provide detailed responses to each reviewer´s comments:

Reviewer 1

This manuscript demonstrates that CD97 overexpression in small intestinal epithelial cells decreases neonatal susceptibility to endotoxin and dampens NF-κB signalling through post-translational stabilization of β-catenin. By interfering with LPS-regulated pathways, this mechanism suppresses p65-dependent pro-inflammatory gene expression. This protective effect is particularly important during early stages of intestinal development, when postnatally acquired negative regulators of TLR4/NF-κB signalling are not fully developed. Overall, the findings indicate that CD97 functions as a protective modulator of intestinal inflammation by stabilizing β-catenin and limiting NF-κB–mediated inflammatory responses. These insights highlight the potential relevance of CD97-mediated signaling in protecting the immature intestine and suggest possible therapeutic implications for neonatal inflammatory disorders such as necrotizing enterocolitis. The experiments are well designed and executed, and addressing a few concerns would further strengthen the study and enhance its impact.

Comments:

Major

Comment 1:

Western blots are shown in several figures; however, densitometric quantification and statistical analysis are lacking. Including these analyses would strengthen the interpretation and significance of the protein expression data.

Response 1:

We thank reviewer 1 for this suggestion and added quantitative densiometric measurements as figures as well as statements within the text. In detail we added a statement within the material and methods section about the quantitative densiometric measurement as follows: “Densiometric quantification of western blot data was performed with Image J (Image J 1.54r).” (Line 242).

Furthermore, we quantified the degree of phosphorylation p65 upon LPS treatment as depicted now in Fig. 4B. We referred to this figure within the text: “Densiometric quantification of phosphorylated p65 revealed a significant difference between WT and TgCD97 after 15 minutes of LPS treatment (Fig. 4B). The overall level of phosphorylated p65 in TgCD97 IECs remained stable throughout the LPS challenge. WT p-p65 levels started to recover back to unstimulated levels at three hours after endotoxin challenge.” (Line 379). Accordingly, we expanded the figure legend: “(B) Densiometric quantification of phosphorylated p65 (S536) related to β-actin over the course of time. Values are depicted as Fold change related to t=0 (n=3).” (Line 414).

Similar, densiometric quantification of β-catenin was performed and added: “Densiometric quantification of β-catenin revealed a significant difference between WT and TgCD97 at 15 minutes of LPS treatment (Fig. 4E).” (Line 401). The figure legend was expanded with: “(D) Densiometric quantification of β-catenin related to β-actin over the course of time. Values are depicted as Fold change related to t=0 (n=3).” (Line 19).

The amount of cytosolic and nuclear β-catenin was also determined and incorporated within the text: “Densiometric quantification of cytosolic and nuclear β-catenin levels revealed a significant difference between WT and TgCD97 derived IECs (Fig. 4G).” (Line 401) as well as within the figure legends: “(G) Densiometric quantification of cytosolic β-catenin between WT and TgCD97 IECs. Cytosolic β-catenin levels were related to GAPDH (n=3). (H) Densiometric quantification of nuclear β-catenin between WT and TgCD97 IECs. Nuclear β-catenin was related to H3 (n=3).” (Line 425)

Comment 2:

In Figure 5C, the GAPDH blots appear blurry and are difficult to interpret. The authors should provide clearer blots for proper evaluation. Alternatively, β-actin could be used as a cytoplasmic loading control for normalization

Response 2:

We thank reviewer 1 for this suggesting and followed his advice by providing a clearer GAPDH signal. At this point we would like to mention that due to figure re-organisation the order of the graph has changed and the GAPDH bot can now be found as Fig. 4F.

Comment 3:

Also, in Figure 5C, two wild-type and three TgCD samples are mentioned in the legends; however, only one wild-type expression band appears to be shown in the blot. The authors should clarify this discrepancy and provide the corresponding data for all the mentioned samples.

Response 3:

We sincerely apologize for this discrepancy. Within the Western Blot analysis we only used one P14 WT mouse as representative. We change this within the figure legends as stated: “(F) Representative western blot for the distribution of β-catenin from one P14 WT and three P14 TgCD97 (TgCD97 #1, #2, #3) within the cytosolic and nuclear fraction.” (Line 423).

Comment 4:

The manuscript would benefit from including concise descriptions of the generation of CD97 overexpressing and knockout mice in the Methods section, to enhance clarity and reproducibility

Response 4:

We thank reviewer 1 for these suggestions and added a detailed description of the generation of the CD97 overexpression and knockout mice in the Methods sections. In detail we added: „ In brief, for the generation of TgCD97 mice, a 9kb regulatory region of the mouse villin gene was place upstream of a CD97 cDNA to generate a villin-CD97 expression construct. Pronuclear microinjection of the purified expression construct into fertilized oocytes of C57BL/6J mice was performed to generate TgCD97 mice. Founders were mated to wild-type mice.” (Line 159). With respect to the generation of CD97 knout-out mice we added: “CD97 knout-out mice were generated using Cre/loxP-mediated DNA recombination. The targeting construct consisted of a loxP-PGKneo-loxP cassette which was inserted upstream of exon 2 and a third loxP site was placed downstream of exon 5 of the murine CD97 gene spanning intron 1 to exon 12. The linearized targeting construct was electroporated into 129/Ola-derived embryonic stem (ES) cells. Selection with G418 was performed for 8 days and G418-resistant ES cell clones were used to PCR based verification of homologous recombination. Subsequently clone IIH10 was microinjected into C57BL/6J-derived blastocytes to generate chimeric offspring which were mated with C57BL/6J mice. Mice positive for germline transmission were mated with homozygous EIIaCre mice expressing Cre recombinase during embryonic development. CD97 deficient mice were backcrossed on a C57BL/6J background.” (Line 163).

Comment 5:

In Figure 3, please include a significance bar comparing treated and untreated CD97 knockout samples to better illustrate their similarity to the wild-type condition. This addition would strengthen the interpretation of whether the treatment mimics the wild-type phenotype

Response 5

We thank Reviewer 1 for highlighting these opportunities for improvement and have added the requested significance bars. As two-way ANOVA accounts for variability across all groups in pairwise comparisons, the differences between wild-type controls and LPS-stimulated IECs did not reach statistical significance. These results further support the similarity between WT and CD97 KO datasets.

Comment 6:

The use of same TNFα and IL-1β values for wild-type mice in both the CD97 overexpression and CD97 knockout experiments is concerning and raises questions regarding the independence of the datasets. The authors must clarify whether the same wild-type control data were used for both experiments and provide a clear justification, as the use of identical control data across separate experiments is generally not acceptable

- Fig 1B and 3A

- Fig 1C and 3B

- Fig 1F and 3C

- Fig 1G and 3D

Response 6:

We thank reviewer 1 for raising the important point and fully agree that transparency regarding data origin is essential.

We would like to clarify that the wild-type (WT) control data used in this study were generated as part of the same experimental series and have been partially reported in a previous publication. The reuse of these WT datasets was intentional, as they serve as a shared baseline control for multiple genetically modified mouse lines analysed under identical experimental conditions (age, tissue, isolation, stimulation protocol and reagents). We chose this approach to ensure direct comparability between CD97 overexpression and CD97 knockout models, while minimizing experimental variability that could arise from independently generated control groups. Importantly, the WT database are not presented as novel datasets in this manuscript. Our main focus and novelty presented in this study lies not in the WT baseline itself, but in the comparative analysis of the CD97 overexpression and knockout as well as the identification of β-catenin mediated regulation of inflammatory signaling in intestinal epithelial cells. To address this concern, we have revised the manuscript and explicitly stated in the methods section that WT control were reused and previously published.

In detail we made several remarks:

“WT control database used in this study were generated as part of the same experimental series and have been partially reported as previously. These data were reused to ensure direct comparability between experimental groups under the same experimental conditions.” (Line 205 - Methods section):

Additionally, we have clearly indicated in the figure legends which data have been reported before.

“WT control data have been partially published previously and are shown here for direct comparison.” (Line 288 Figure 1 - Legend):

“WT control data shown in this figure are identical to those presented in Figure 1 and were derived from the same experimental dataset which have been partially published previously and are shown here for direct comparison.” (Line 334 Figure 3 Legend):

We also provided the raw data in the supplementary material.

We believe that this transparent approach ensures scientific rigor while allowing robust comparison across experimental conditions.

We would like to emphasize that we contacted PLOS One with respect to the WT database and attached the original message from our side:

--------------- Original Message ---------------

From: Niklas Dressler [em@editorialmanager.com]

Sent: 2/6/2026, 3:14 AM

To: plosone@plos.org

Subject: Author Query to Editor PONE-D-26-04060; Disclosure of Previously Published Partial Dataset - [EMID:d20cf7804f916212]

EXTERNAL EMAIL – SECURITY NOTICE

This message originated outside of PLOS. Exercise caution when interacting with links, attachments, or requests for sensitive information.

EXTERNAL EMAIL – SECURITY NOTICE

This message originated outside of PLOS. Exercise caution when interacting with links, attachments, or requests for sensitive information.

Manuscript information:

========================

PONE-D-26-04060

Overexpression of CD97 in intestinal epithelial cells attenuates LPS-induced pro-inflammatory cytokine induction via stabilization of β-catenin early in life

PLOS One

========================

Dear Editors,

thank you very much for your time and for handling our submitted manuscript.

We would like to inform you that, contrary to our initial information during submission, a small part of the dataset has already been published in a separate article. This concerns the raw qRT-PCR data of TNF-α expression in wild-type intestinal epithelial cells with and without LPS stimulation.

These data were generated in parallel with another project investigating age-dependent inflammatory mediator patterns in intestinal epithelial cells and the coordinated response of intraepithelial lymphocytes to endotoxin stimulation. The corresponding article was published in Paediatric Research on July 21, 2025:

Murine intestinal epithelial cells and lymphocytes undergo contrasting inflammatory shifts during gastrointestinal development

https://doi.org/10.1038/s41390-025-04262-z

The relevant data are presented in Figure 3A of that publication and correspond to Figure 1A–D in our submitted manuscript, where the red bars represent the previously published dataset.

We would like to emphasize that the primary focus of the present manuscript is the comparative analysis between wild-type and CD97-overexpressing intestinal epithelial cells across different developmental stages. Therefore, the scientific scope, research questions, and main conclusions of the two studies differ substantially.

We sincerely apologize for this oversight and for any inconvenience it may cause. We fully appreciate the importance of transparency and would be grateful for your guidance on how to proceed.

Thank you very much for your understanding and consideration.

Kind regards,

Niklas Dressler

on behalf of the authors

Comment 7:

The novelty of the study is not well established. Multiple previous studies have already demonstrated the role of CD97 following LPS induction; therefore, the authors must clearly define the novel aspect of this work and explain how it advances current understanding beyond what is already known.

Response 7:

We thank reviewer 1 for this important comment. We agree that CD97 has previously been implicated in the regulation of inflammatory responses following LPS stimulation, particulary in macrophages and in models of colonic inflammation. However, the present study provides several novel aspects that extend current understanding in a biologically and clinically relevant manner:

• First characterization of CD97 function in immature small intestinal epithelium

Previous studies have primarily focused on immune cells (e.g. macrophages) or adult colonic tissue. To our knowledge, this is the first study investigating CD97 function specifically in primary small intestinal epithelial cells (IECs) across defined postnatal developmental stages that model human neonatal gut maturation.

• Demonstration of an age-dependent developmentally restricted effect

We show that CD07-mediated attenuation of LPS-induced inflammation is restricted to early developmental stages (P7-P28), while adult IECs are largely unresponsive. This identifies CD97 as a regulator of developmental endotoxin sensitivity, which has not been previously described.

• Tissue-specific effect in the ileum

Our data demonstrates that the anti-inflammatory effect of CD97 is restricted to ileal tissue, the primary site of necrotizing enterocolitis (NEC). This spatial specificity has been reported before and provides translational relevance to neonatal intestinal disease.

• Functional validation via pharmacological inhibition of β-catenin

By using the inhibitor iCRT3, we demonstrate that blocking β-catenin signaling restores the pro-inflammatory response in CD97-overexpressing IECs. This provides causal evidence linking β-catenin stabilization to the observed phenotype.

• Relevance to neonatal disease mechanisms (NEC)

By integrating developmental timing, ileal specificity, and β-catenin/NF-κB crosstalk, our study provided a mechanistic framework linking CD97 to endotoxin tolerance in the immature gut, which is highly relevant to NEC pathogenesis.

To address these novelties within the manuscript we made several changes within the manuscript.

• “This effect was restricted to e

Attachment Submitted filename: Response to Reviewers.docx

# Peer review: Decision Letter 1

**DOI:** 10.1371/journal.pone.0354507.r003

**Authors:** Pradeep Dudeja

10 Jul 2026

Overexpression of CD97 in intestinal epithelial cells attenuates LPS-induced pro-inflammatory cytokine induction via stabilization of β-catenin early in life

PONE-D-26-04060R1

Dear Dr. Dressler,

We’re pleased to inform you that your manuscript has been judged scientifically suitable for publication and will be formally accepted for publication once it meets all outstanding technical requirements.

Within one week, you’ll receive an e-mail detailing the required amendments. When these have been addressed, you’ll receive a formal acceptance letter and your manuscript will be scheduled for publication.

An invoice will be generated when your article is formally accepted. Please note, if your institution has a publishing partnership with PLOS and your article meets the relevant criteria, all or part of your publication costs will be covered. Please make sure your user information is up-to-date by logging into Editorial Manager at Editorial Manager® and clicking the ‘Update My Information' link at the top of the page. For questions related to billing, please contact billing support.

If your institution or institutions have a press office, please notify them about your upcoming paper to help maximize its impact. If they’ll be preparing press materials, please inform our press team as soon as possible -- no later than 48 hours after receiving the formal acceptance. Your manuscript will remain under strict press embargo until 2 pm Eastern Time on the date of publication. For more information, please contact onepress@plos.org.

Kind regards,

Pradeep Dudeja

Academic Editor

PLOS One

Additional Editor Comments (optional):

Reviewers' comments:

Reviewer's Responses to Questions

**Comments to the Author**

1. If the authors have adequately addressed your comments raised in a previous round of review and you feel that this manuscript is now acceptable for publication, you may indicate that here to bypass the “Comments to the Author” section, enter your conflict of interest statement in the “Confidential to Editor” section, and submit your "Accept" recommendation.

Reviewer #1: All comments have been addressed

Reviewer #2: All comments have been addressed

**********

2. Is the manuscript technically sound, and do the data support the conclusions?

The manuscript must describe a technically sound piece of scientific research with data that supports the conclusions. Experiments must have been conducted rigorously, with appropriate controls, replication, and sample sizes. The conclusions must be drawn appropriately based on the data presented.

Reviewer #1: Yes

Reviewer #2: Yes

**********

3. Has the statistical analysis been performed appropriately and rigorously?

Reviewer #1: Yes

Reviewer #2: I Don't Know

**********

4. Have the authors made all data underlying the findings in their manuscript fully available?

The PLOS Data policy requires authors to make all data underlying the findings described in their manuscript fully available without restriction, with rare exception (please refer to the Data Availability Statement in the manuscript PDF file). The data should be provided as part of the manuscript or its supporting information, or deposited to a public repository. For example, in addition to summary statistics, the data points behind means, medians and variance measures should be available. If there are restrictions on publicly sharing data—e.g. participant privacy or use of data from a third party—those must be specified.

Reviewer #1: Yes

Reviewer #2: Yes

**********

5. Is the manuscript presented in an intelligible fashion and written in standard English?

PLOS ONE does not copyedit accepted manuscripts, so the language in submitted articles must be clear, correct, and unambiguous. Any typographical or grammatical errors should be corrected at revision, so please note any specific errors here.

Reviewer #1: Yes

Reviewer #2: Yes

**********

6. Review Comments to the Author

Please use the space provided to explain your answers to the questions above. You may also include additional comments for the author, including concerns about dual publication, research ethics, or publication ethics. (Please upload your review as an attachment if it exceeds 20,000 characters)

Reviewer #1: The authors have adequately addressed all the concerns raised by the reviewers, and the manuscript has improved substantially. It is now in good shape for publication. I noted only one minor issue: the word "Densitometric" appears to contain a spelling error throughout the manuscript and should be corrected consistently. Other than this minor typographical issue, the manuscript is in good shape.

Reviewer #2: (No Response)

**********

7. PLOS authors have the option to publish the peer review history of their article (what does this mean?). If published, this will include your full peer review and any attached files.

If you choose “no”, your identity will remain anonymous but your review may still be made public.

**Do you want your identity to be public for this peer review?**  For information about this choice, including consent withdrawal, please see our Privacy Policy.

Reviewer #1: **Yes:**  Nazim Husain

Reviewer #2: **Yes:**  Anoop Kumar

**********

# Editor report: Acceptance letter

**DOI:** 10.1371/journal.pone.0354507.r004

**Authors:** Pradeep Dudeja

PONE-D-26-04060R1

PLOS One

Dear Dr. Dressler,

I'm pleased to inform you that your manuscript has been deemed suitable for publication in PLOS One. Congratulations! Your manuscript is now being handed over to our production team.

At this stage, our production department will prepare your paper for publication. This includes ensuring the following:

* All references, tables, and figures are properly cited

* All relevant supporting information is included in the manuscript submission,

* There are no issues that prevent the paper from being properly typeset

You will receive further instructions from the production team, including instructions on how to review your proof when it is ready. Please keep in mind that we are working through a large volume of accepted articles, so please give us a few days to review your paper and let you know the next and final steps.

Lastly, if your institution or institutions have a press office, please let them know about your upcoming paper now to help maximize its impact. If they'll be preparing press materials, please inform our press team within the next 48 hours. Your manuscript will remain under strict press embargo until 2 pm Eastern Time on the date of publication. For more information, please contact onepress@plos.org.

You will receive an invoice from PLOS for your publication fee after your manuscript has reached the completed accept phase. If you receive an email requesting payment before acceptance or for any other service, this may be a phishing scheme. Learn how to identify phishing emails and protect your accounts at https://explore.plos.org/phishing.

If we can help with anything else, please email us at customercare@plos.org.

Thank you for submitting your work to PLOS ONE and supporting open access.

Kind regards,

PLOS ONE Editorial Office Staff

on behalf of

Dr. Pradeep Dudeja

Academic Editor

PLOS One
